# An Environmental Stable Ionogel‐Based Smart Glove System for Integrated Underwater Communication and Safety Warning

**DOI:** 10.1002/advs.78325

**Published:** 2026-10-28

**Authors:** Xiaoqin Li, Jianwen Liu, Wenhao Yang, Wenjing Yue, Nam‐Young Kim, Young‐Kee Shin, Song Gao, Eun‐Seong Kim, Yang Li

**Affiliations:** ^1^ Shandong Key Laboratory of Next‐Generation Semiconductor Technology and Systems School of Integrated Circuits Shandong University Jinan China; ^2^ Shandong Provincial Key Laboratory of Ubiquitous Intelligent Computing School of Information Science and Engineering University of Jinan Jinan China; ^3^ RF Bio Center Department of Electronics Engineering Kwangwoon University Seoul Republic of Korea; ^4^ Laboratory of Molecular Pathology and Cancer Genomics Department of Molecular Medicine and Biopharmaceutical Sciences Graduate School of Convergence Science and Technology Seoul National University Seoul Republic of Korea; ^5^ Department of Medical and Digital Engineering College of Engineering Hanyang University Seoul South Korea

**Keywords:** environmental stabilities, flexible materials, soft sensing, underwater communication, wearable sensors

## Abstract

Flexible sensing technology based on hydrogel‐based smart gloves enables accurate gesture recognition in complex environments, but such gloves degrade in dry, high‐temperature, and underwater conditions, and their complex assembly limits practical usability. Here, a smart glove monitoring system is developed by integrating a poly(benzyl methacrylate‐co‐2‐hydroxyethyl acrylate)/ionic liquid (P(BZMA‐co‐HEA)/IL) ionogel sensing layer with a flexible printed circuit board (FPCB). The ionogel is constructed through a network‐constrained ionic soft‐space regulation strategy, where HEA‐rich segments strengthen the network, IL provides deformation‐adaptive ionic soft spaces, and BZMA‐rich hydrophobic segments suppress water penetration. The optimized ionogel combines stretchability (up to 1100%), weight retention (98.86% at room temperature and 95.25% at 65°C), favorable adhesion, anti‐swelling behavior, and stable sensing performance under multiple environmental conditions. The FPCB enables a detachable and reconfigurable sensor array, ensuring stable interfacial contact and wearing flexibility. By further combining multi‐channel signal acquisition, fully connected neural network (FCNN)‐assisted gesture classification, and real‐time alert feedback, the smart glove functions as an integrated sensing‐recognition‐warning platform, achieving 99.92% gesture recognition accuracy and enabling reliable underwater communication and safety warning. This work establishes an effective material design and integration strategy for environmentally adaptive smart gloves.

## Graphical abstract

This study develops a smart glove by integrating a P(BZMA‐co‐HEA)/IL ionogel with a reconfigurable FPCB for underwater communication and real‐time safety warning. The network‐constrained ionic soft‐space regulation strategy enables mechanical reliability, environmental stability, and sensing adaptability, while the modular FPCB sensor array simplifies assembly and improves device reliability.

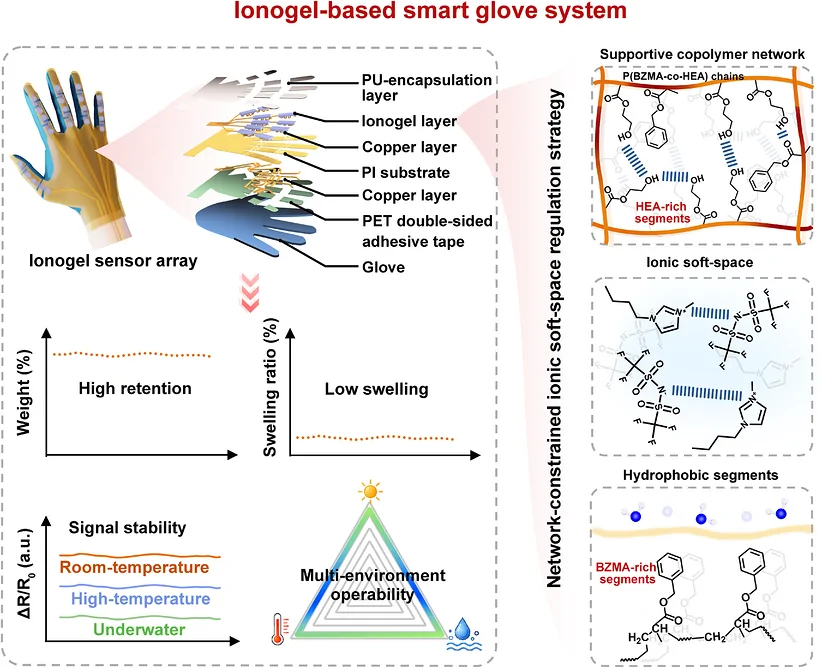

## 1 Introduction

The rapid advancement of flexible sensing technology has significantly driven innovative breakthroughs in wearable devices across fields, such as movement monitoring [1, 2], human‐computer interaction interfaces [3, 4], and specialized work equipment [5, 6]. Among these, smart gloves stand out as a key application. By integrating flexible sensors at the finger joints, these gloves capture real‐time hand movements and translate them into continuous electrical signals [7, 8]. This process enables efficient motion recognition and command transmission in complex scenarios such as virtual reality control [9, 10], remote interaction [11, 12], and medical healthcare [13, 14]. Hydrogels have been widely explored as flexible sensing materials owing to their tissue‐like softness, stretchability, and ionic conductivity [15, 16]. Nevertheless, their water‐dependent networks lead to poor environmental adaptability. Water evaporation under dry or high‐temperature conditions causes shrinkage, hardening, and signal distortion, whereas water uptake in aqueous environments induces swelling, mechanical deterioration, and unstable sensing signals [17]. To improve the environmental stability of hydrogels, previous studies have introduced polyols [18], inorganic salts [19], or zwitterionic molecules [20] to suppress water evaporation, while crosslinking‐density regulation [21], solvent exchange [22], or hydrophobic segment incorporation [23] have been used to reduce swelling. However, traditional hydrogel systems still struggle to simultaneously achieve dehydration resistance, anti‐swelling capability, thermal stability, mechanical compliance, and long‐term sensing reliability.

Ionogels, which employ ionic liquids (ILs) as dispersion media, provide a promising alternative to overcome these limitations. Owing to their negligible vapor pressure, excellent thermal stability, strong solvation capability, and stable ionic conductivity, ILs can avoid dehydration‐induced failure while maintaining softness and ion transport capability [24, 25]. Moreover, ILs show good compatibility with various hydrophobic monomers, enabling the construction of water‐resistant polymer networks without the severe phase incompatibility commonly encountered in aqueous hydrogel systems [26, 27]. Therefore, ionogels have been widely explored for flexible sensing, soft electronics, and human‐machine interfaces [28, 29, 30]. In wet or underwater environments, water uptake, interfacial interference, or ion loss may deteriorate the mechanical integrity and sensing reliability of ionogels. To address wet‐environment instability, recent strategies based on fluorinated polymer networks, supramolecular coupling, and amphiphilic phase‐separated structures have improved the underwater stability and anti‐swelling performance of ionogels, enabling their use in underwater sensing and anti‐swelling flexible electronics [31, 32, 33]. Nevertheless, thermally induced gel‐sol transition in some ionogel systems can still lead to network failure and unstable sensing output [34]. In practical applications, ionogel design is still limited by intrinsic trade‐offs among mechanical adaptability, swelling resistance, dehydration tolerance, thermal stability, ion‐transport stability, and device‐level integration reliability. This issue becomes especially critical for smart glove applications, where sensing units must withstand repeated finger‐joint deformation and maintain stable electrical signals under multi‐environmental conditions. Moreover, conventional smart gloves usually rely on manually soldered flying wires to connect sensing units with the back‐end circuit, which increases assembly complexity and may cause signal instability during repeated motion or water exposure. Therefore, it is highly desirable to develop a multi‐environment‐stable ionogel and integrate it with a reliable flexible circuit platform for robust smart glove sensing applications.

In this work, we develop a multi‐environment‐operable ionogel‐based smart glove system based on a network‐constrained ionic soft‐space regulation strategy, aiming to overcome the environmental instability and complicated assembly of conventional hydrogel‐based smart gloves (Figure 1a). Unlike sensing systems that operate reliably only under a single condition, the present smart glove is designed as a “tri‐environment‐stable” platform, maintaining stable sensing responses under room‐temperature, high‐temperature, and underwater conditions. The key material design lies in the construction of a poly(benzyl methacrylate‐co‐2‐hydroxyethyl acrylate)/ionic liquid (P(BZMA‐co‐HEA)/IL) ionogel network with synergistic structural regulation. In this network, HEA‐rich segments serve as a supportive polymer framework through dynamic interactions, thereby enhancing network constraint. Meanwhile, the IL, 1‐butyl‐3‐methylimidazolium bis(trifluoromethanesulfonyl)imide ([BMIM][TFSI]), constructs adaptive ionic soft spaces between polymer chains to promote chain mobility and deformation compliance. In addition, BZMA‐rich hydrophobic segments help suppress water penetration and stabilize the polymer network under humid or aqueous environments (Figure 1b). This cooperative structural regulation endows the ionogel with high stretchability up to 1100%, high weight retention of 98.86% at room temperature and 95.25% at 65°C, favorable adhesion, excellent anti‐swelling capability, and stable sensing performance under multiple environmental conditions. Integrated with a flexible printed circuit board (FPCB), the ionogel sensing units enable detachable and reconfigurable sensor assembly, thereby simplifying the conventional fabrication process and improving structural flexibility. By coupling multi‐channel signal acquisition,fully connected neurral network (FCNN)‐assisted gesture recognition, and real‐time alert feedback, the smart glove establishes a sensing‐recognition‐warning platform for underwater human‐machine interaction, achieving 99.92% gesture recognition accuracy and reliable safety warning feedback underwater.

**FIGURE 1:**
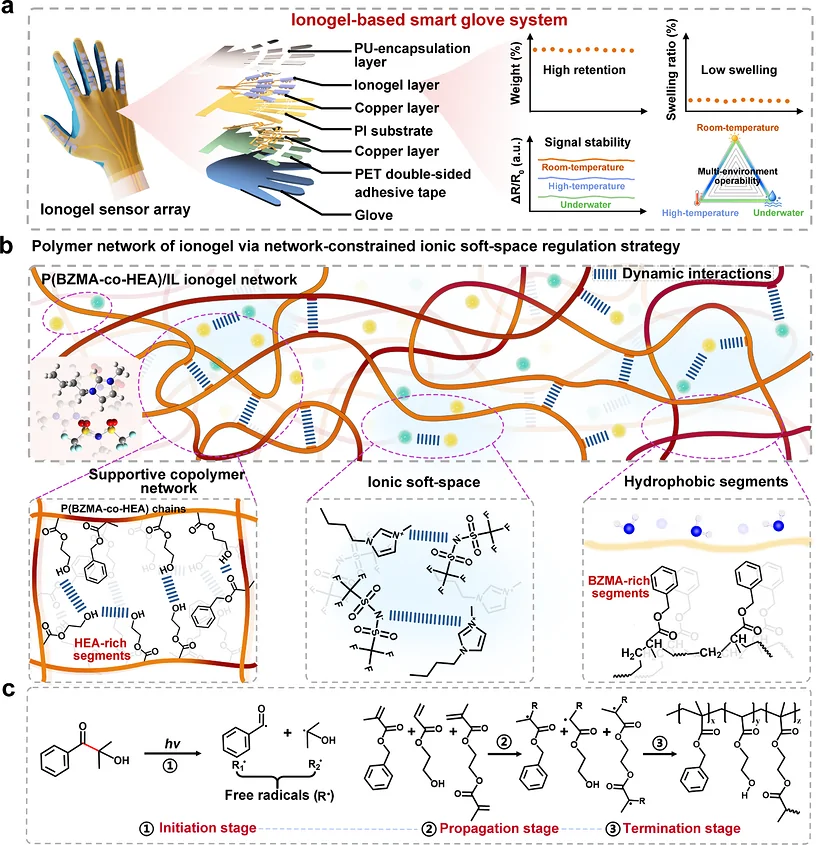
Schematic illustration of the ionogel‐based smart glove and its material design strategy. (a) Schematic illustration of the detachable FPCB‐integrated smart glove constructed from P(BZMA‐co‐HEA)/IL ionogel sensing units. The diagram highlights the modular sensor‐array architecture and the intrinsic tri‐environment stability of the ionogel sensing layer under room‐temperature, high‐temperature, and underwater conditions. (b) Network‐constrained ionic soft‐space regulation strategy of the P(BZMA‐co‐HEA)/IL ionogel. The HEA‐rich supportive network enhances structural constraint, [BMIM][TFSI] forms adaptive ionic soft spaces to improve chain mobility and deformation compliance, and BZMA‐rich hydrophobic domains suppress water penetration, enabling stable sensing performance across multiple environments. (c) Schematic illustration of the UV‐initiated free‐radical polymerization process.

## 2 Results and Discussion

### 2.1 Design and Fabrication of the Smart Glove

As shown in Figure 1a, this study presents an ionogel‐based smart glove system for the real‐time tracking of divers’ hand movements in underwater environments. The multilayer structure of the device consists sequentially of a glove substrate, a PET double‐sided adhesive layer, an FPCB, ionogel active layers, and a polyurethane (PU) encapsulation layer. The FPCB, fabricated on a polyimide (PI) flexible substrate with a dual conductive layer configuration, provides a stable sensing signal transmission platform for the system. A key feature of this design is the introduction of a highly integrated FPCB architecture into the smart glove platform, effectively mitigating signal instability caused by electrode exposure and water infiltration under underwater conditions. Specifically, a conformal and stable contact interface is formed between the FPCB electrodes and the ionogel sensing layer, ensuring efficient signal transmission during repeated finger deformation. Meanwhile, the inherent encapsulation structure of the FPCB suppresses external water penetration, thereby improving environmental reliability. Combined with a custom‐developed signal acquisition module, the smart glove simultaneously captures motion signals from ten finger joints. When finger bending occurs, deformation of the ionogel sensing units generates real‐time sensing signal variations, which are wirelessly transmitted to a receiving terminal, enabling continuous motion tracking and gesture recognition. Compared with conventional smart glove fabrication methods requiring complex wiring processes, the synergistic integration of FPCB and ionogel significantly simplifies system assembly. The FPCB (Figure S1a) exhibits excellent flexibility and structural adaptability, allowing stable attachment to curved glove surfaces. Benefiting from the excellent flexibility and conformal capability of the ionogel, the sensing layers maintain stable attachment to the glove even under repeated bending deformation (Figure S1b), ensuring reliable signal acquisition. In addition, the detachable design of the ionogel components enables rapid replacement and reconfiguration, improving system maintainability and adaptability.

At the material level, the ionogel sensing layer is synthesized via UV‐induced in situ copolymerization of BZMA and HEA in the IL, forming a P(BZMA‐co‐HEA)/IL ionogel with multiple intermolecular interactions. Compared with the precursor solution, the resulting ionogel exhibits markedly increased viscosity over the measured frequency range, confirming the formation of a crosslinked network with enhanced viscoelasticity and structural stability (Figure S2). The polymerization of P(BZMA‐co‐HEA) follows a typical free‐radical reaction mechanism, comprising three stages (Figure 1c). During the photoinitiation stage, UV irradiation induces homolytic cleavage of Irgacure 1173 molecules, generating reactive benzoyl radicals (R1·) and isopropanol radicals (R2·). Subsequently, in the chain propagation stage, these radicals undergo sequential addition reactions with unsaturated bonds of monomers, promoting continuous elongation of polymer chains. Finally, in the termination stage, the active radicals are quenched by the combination of two kinetic chains, forming a chemically crosslinked P(BZMA‐co‐HEA) network structure. The P(BZMA‐co‐HEA)/IL ionogel exhibits diverse intermolecular and intramolecular interactions, mainly including covalent crosslinking networks, hydrogen bonds, and ion‐dipole interactions. These synergistic interactions collectively endow the ionogel with excellent mechanical strength, water retention, high‐temperature tolerance, self‐adhesion, and anti‐swelling properties, thereby ensuring the stable operation of the smart glove in complex environments.

### 2.2 Mechanical Properties and Characterization of Ionogel Active Layers

This study systematically investigates the mechanical properties of P(BZMA‐co‐HEA)/IL ionogel as an active layer for smart gloves. As shown in Figure 2a, the introduction of the HEA monomer is essential for the formation of a stable gel network, as no obvious gelation is observed in the absence of HEA. Fourier transform infrared (FTIR) spectroscopy is subsequently employed to characterize network formation and component integration. As shown in Figure 2b, the characteristic C═C peak at 1635 cm^−1^ associated with HEA and BZMA monomers disappears after polymerization, confirming the successful opening of the double bonds and the formation of a cross‐linked network [35]. In the spectrum of the P(BZMA‐co‐HEA)/IL ionogel, the C═O stretching vibration at 1726 cm^−1^ and the characteristic benzene ring vibrations at 740 and 701 cm^−1^ confirm successful incorporation of BZMA units [36]. Moreover, the C═N stretching vibration of the imidazolium ring at 1570 cm^−1^ and the appearance of the SO_2_ absorption peaks at 1349 and 1327 cm^−1^, consistent with those of the pure IL, verify successful introduction of the IL [37, 38, 39]. Notably, the ─OH stretching vibration shifts from 3427 cm^−1^ in the HEA monomer to 3419 cm^−1^ in the P(BZMA‐co‐HEA) gel, indicating strengthened hydrogen‐bonding interactions within the polymer network [40]. A further shift to 3525 cm^−1^ in the ionogel suggests that incorporation of the IL significantly modifies the hydrogen‐bonding environment. These results confirm successful construction of the target ionogel network. In addition, the ionogel exhibits excellent transparency, with an average transmittance of ≈90% in the 400–800 nm range (Figure 2c), highlighting its potential for applications requiring optical compatibility. The mechanical performance of the ionogel active layer is systematically evaluated using a universal testing machine. The tensile stress–strain curves of ionogels with varying HEA content are presented in Figure 2d (all samples with 33% [BMIM][TFSI]). As the HEA content increases from 35% to 60%, the tensile strength, elastic modulus, and toughness increase from 127.86 kPa, 13.07 kPa, and 668.55 kJ m^−3^ to 299.83 kPa, 99.12 kPa, and 1312.26 kJ m^−3^ (Figure S3a and Figure 2f), respectively, while the elongation at break decreases from 1304.46% to 875.22%. These results indicate that the introduction of HEA monomers effectively regulates the mechanical strength of the ionogel by increasing the proportion of supportive segments within the copolymer network [41]. This synergistic effect contributes to maintaining the structural stability of smart gloves under repeated deformation. In contrast, the IL content markedly influences the deformability of the ionogel active layer. Figure 2e displays the stress–strain curves of ionogels with varying [BMIM][TFSI] contents (all samples with 50% HEA). As the [BMIM][TFSI] content increases from 22% to 44%, the elongation at break increases from 764.04% to 1146.50%, while the tensile strength, elastic modulus, and toughness decrease from 431.29 kPa, 138.27 kPa, and 1580.21 kJ m^−3^ to 141.48 kPa, 45.52 kPa, and 877.53 kJ m^−3^ (Figure S3b and Figure 2g), respectively. This behavior is attributed to the plasticizing effect of the IL [42, 43, 44, 45]. Consequently, considering the mechanical reliability and deformation adaptability required for smart glove applications, the formulation containing 50% HEA and 33% IL is selected for subsequent performance evaluation.

**FIGURE 2:**
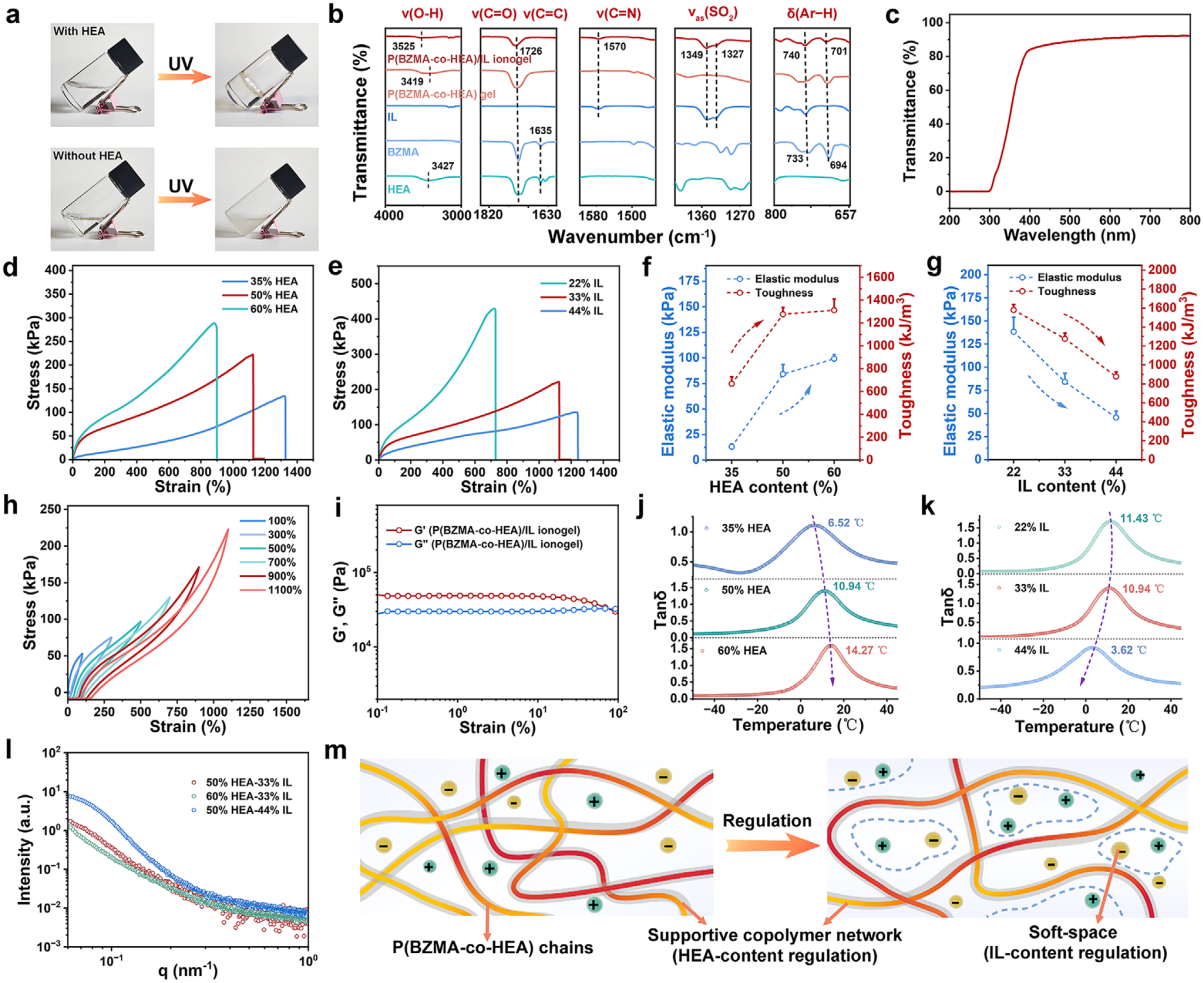
Mechanical properties and characterization of the ionogel active layer. (a) Gel behavior with and without HEA. (b) FT–IR spectra of HEA, BZMA, IL, P(BZMA‐co‐HEA) gel, and P(BZMA‐co‐HEA)/IL ionogel. (c) UV–vis spectra of the P(BZMA‐co‐HEA)/IL ionogel. (d) Stress–strain curves with different HEA contents. (e) Stress–strain curves with different IL contents. (f) Elastic modulus and toughness of ionogels with different HEA contents. (g) Elastic modulus and toughness of ionogels with different IL contents. (h) Loading–unloading cycles of the P(BZMA‐co‐HEA)/IL ionogel under 100% to 1100% tensile strain. (i) Modulus of the P(BZMA‐co‐HEA)/IL ionogel under strain amplitude sweep. (j) Tanδ‐temperature curves of ionogels with different HEA contents. (k) Tanδ‐temperature curves of ionogels with different IL contents. (l) One‐dimensional SAXS profiles of ionogels with different HEA and IL contents. (m) Schematic illustration of the network‐constrained ionic soft‐space regulation strategy.

As shown in Figure S4a,b, the prepared P(BZMA‐co‐HEA)/IL ionogel sustains tensile strains of up to ≈1100% and supports a 700 g load without fracture, demonstrating excellent mechanical reliability for wearable glove applications. To further evaluate the deformability and resilience of the ionogel, cyclic loading–unloading tests are performed over a strain range of 100%–1100% (Figure 2h). The stress–strain curves exhibit pronounced hysteresis loops with increasing areas at higher strains, indicating enhanced energy dissipation arising from reversible noncovalent interactions and gradual polymer chain disentanglement within the network. Despite the observed hysteresis, the ionogel exhibits readily mechanical recovery across the entire strain range, confirming its high elasticity. Rheological measurements further reveal the viscoelastic characteristics of the ionogel. During strain amplitude sweep tests, the storage modulus (G′) remains higher than the loss modulus (G″) up to a critical strain of 68.95% (Figure 2i), indicating that the network structure remains intact within this deformation regime. The frequency sweep test shows that G′ is consistently higher than G″ within 0.1–10 Hz, confirming the elastic‐dominated solid‐like viscoelastic behavior of the ionogel (Figure S5a). To further evaluate the self‐recovery capability of the ionogel network under dynamic deformation, step‐strain time sweep measurements are conducted by periodically switching the applied strain between 0.1% and 67%. As shown in Figure S5b, G′ remains higher than G″ throughout the strain‐switching process, indicating that the gel network maintains structural integrity under repeated deformation. Moreover, both G′ and G″ recover to stable plateau values after each strain transition without obvious modulus decay, demonstrating the excellent self‐recovery ability and viscoelastic stability of the ionogel.

In order to better elucidate the roles of copolymer‐network confinement and IL plasticization in regulating the mechanical properties of the ionogels, dynamic mechanical analysis (DMA) temperature‐sweep tests are performed on P(BZMA‐co‐HEA)/IL ionogels with different compositions to investigate the thermomechanical behavior with increasing temperature. As shown in Figure S6a, all samples exhibit typical glassy, glass‐transition, and elastic regions. With increasing temperature, the storage modulus E′ gradually decreases, indicating that thermal stimulation weakens the dynamic interactions within the polymer network and promotes thermally activated segmental relaxation (Figure S6b). With increasing HEA content from 35% to 60%, the tanδ peak shifts from 6.52°C to 14.27°C (Figure 2j), and the temperature corresponding to the maximum E′ decreasing rate increases from approximately 4.5°C to 10°C. These results indicate that increasing the HEA content enhances the supportive structural units and segmental confinement within the network, enabling more effective stress bearing and suppressing excessive chain motion. In contrast, the IL content mainly regulates the plasticization degree of the network and the chain‐slippage capability. As shown in Figure 2k, the tanδ peak of the ionogel with 22% IL is located at 11.43°C, indicating stronger network constraint at a lower IL content. Therefore, this ionogel shows higher mechanical strength but limited stretchability. When the IL content further increases to 44%, the tanδ peak significantly decreases to 3.62°C, and the temperature corresponding to the maximum E′ decreasing rate shifts forward to approximately −0.92°C. This indicates that excessive IL enhances the plasticization effect, allowing polymer chains to undergo thermally activated slippage and viscoelastic softening at a lower temperature. Although the permanent crosslinked framework still maintains the overall network integrity, excessive IL weakens the effective supporting capability of the network. Small‐angle X‐ray scattering (SAXS) analysis is further performed to investigate the influence of HEA and IL contents on the network structure of the ionogels. The 2D SAXS patterns show nearly isotropic scattering distributions for all three samples (Figure S7). The 1D SAXS profiles (Figure 2l) show that increasing the HEA content from 50% to 60% at a fixed IL content of 33% decreases the low‐q (*q* = 0.06028 nm^−1^) scattering intensity from 1.75 to 1.51 a.u., indicating reduced electron‐density fluctuation and a more homogeneous ionogel network [46]. In contrast, increasing the IL content to 44% markedly enhances the intensity to 7.94 a.u. at the same q, approximately 4.54 times that of the 50% HEA‐33% IL ionogel. Combined with the DMA results, the enhanced low‐q scattering indicates that excessive IL introduces stronger local compositional fluctuations and soft‐phase structures. The low‐q broad shoulder in the I(q)*q^2^‐q curve corresponds to an apparent characteristic length of approximately 89 nm, suggesting the formation of local ionic soft spaces between polymer chains (Figure S8) [47]. Therefore, tuning the supportive copolymer network and the ionic soft space enables a balance between stability and deformation compliance of the ionogel network (Figure 2m).

### 2.3 Temperature Tolerance and Adhesion Properties of Ionogel Active Layers

The moisture‐retention capability and thermal tolerance of the ionogel active layer are critical for long‐term operation of smart gloves. To evaluate environmental stability, the weight retention and mechanical properties of the ionogel are measured after storage at room temperature (RT) and 65°C. As shown in Figure 3a, the ionogel maintains high weight retention rates of 98.86% at RT and 95.25% at 65°C after 30 days, with negligible changes in appearance or dimensions. Thermogravimetric analysis (TGA) reveals a thermal decomposition temperature of 326°C under a nitrogen atmosphere, accompanied by minimal mass loss (≈3%). Moreover, the higher residual mass of the P(BZMA‐co‐HEA)/IL ionogel compared with the P(BZMA‐co‐HEA) gel further confirms its improved thermal stability (Figure 3b). Figure 3c shows that the ionogel retains excellent mechanical performance after 30 days of storage at RT. Even after storage at 65°C for 20 days, the ionogel maintains a tensile strength of 285 kPa and an elongation at break of 911.72%, while preserving excellent flexibility after 30 days. As visually confirmed in Figure S9, the ionogel exhibits outstanding stretchability under both storage conditions. Correspondingly, Rheological temperature‐sweep results (Figure 3d) show that G′ consistently exceeds G″ over the temperature range of 26.39°C–79.48°C, indicating preservation of a stable gel state across this interval. These results demonstrate that the ionogel active layer maintains robust mechanical integrity and environmental stability during long‐term operation in complex conditions, which is essential for reliable and durable smart glove applications.

**FIGURE 3:**
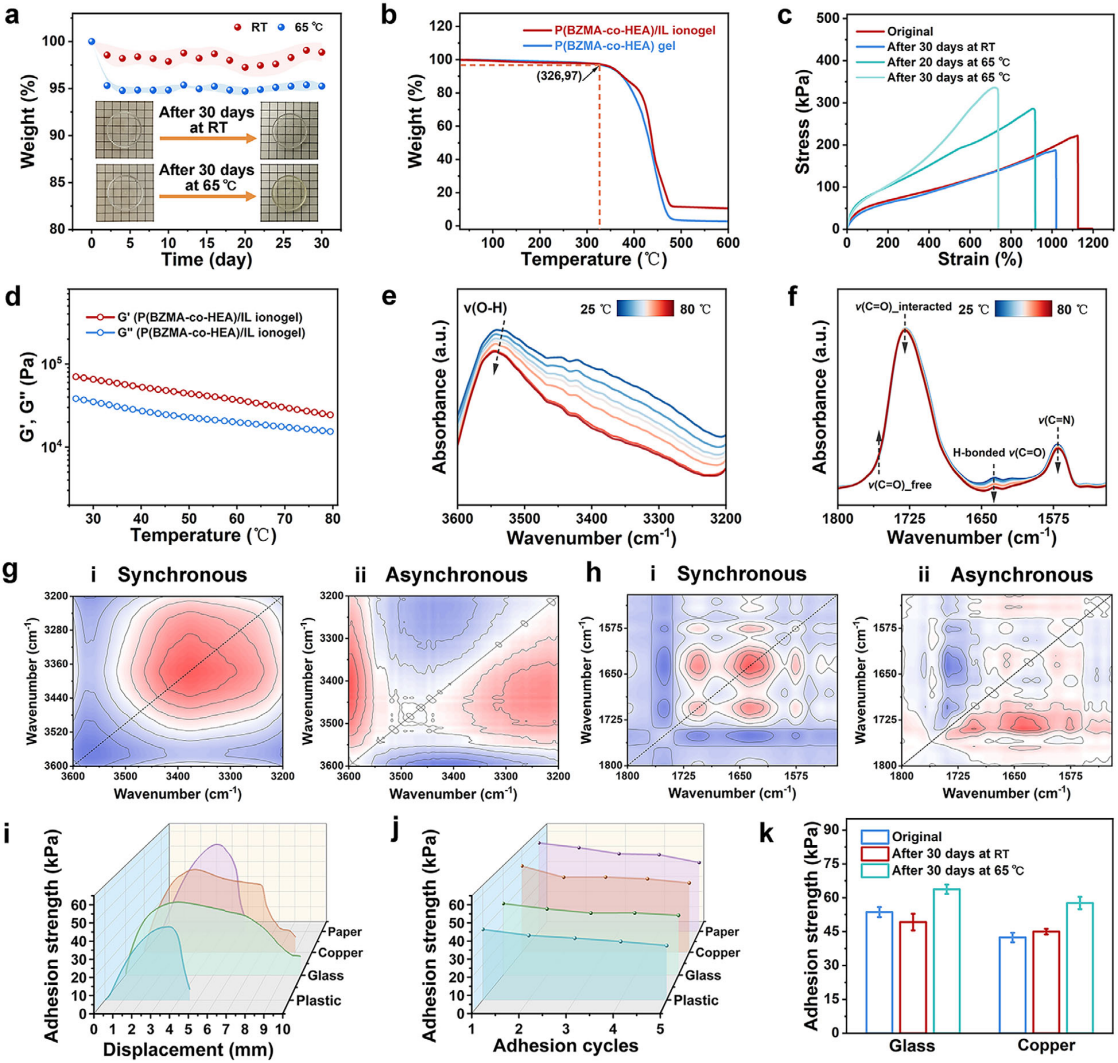
Thermal tolerance and adhesion properties of the ionogel active layer. (a) Weight retention ratios of the P(BZMA‐co‐HEA)/IL ionogel stored at RT and 65°C for 30 days. (b) TGA curves of the P(BZMA‐co‐HEA)/IL ionogel and P(BZMA‐co‐HEA) gel. (c) Stress–strain curves of the P(BZMA‐co‐HEA)/IL ionogel at different temperatures and storage times. (d) The modulus of P(BZMA‐co‐HEA)/IL ionogel at 26.39°C–79.48°C. (e) Temperature‐dependent ATR‐FTIR spectra of the P(BZMA‐co‐HEA)/IL ionogel in the 3200–3600 cm^−1^ range. (f) Temperature‐dependent ATR‐FTIR spectra of the P(BZMA‐co‐HEA)/IL ionogel in the 1520–1800 cm^−1^ range. (g) Synchronous and asynchronous 2D correlation spectra generated from the 3200–3600 cm^−1^ region. (h) Synchronous and asynchronous 2D correlation spectra generated from the 1520–1800 cm^−1^ region. Red and blue represent positive and negative correlation signals, respectively. (i) Lap‐shear strength curves of P(BZMA‐co‐HEA)/IL ionogel on different substrates. (j) P(BZMA‐co‐HEA)/IL ionogel adhesion strength on different substrates over 5 cycles. (k) P(BZMA‐co‐HEA)/IL ionogel adhesion strength on glass and copper substrates after storage at RT and 65°C for 30 days.

To elucidate thermally induced molecular interactions and structural evolution, temperature‐dependent ATR‐FTIR spectroscopy and two‐dimensional correlation spectroscopy (2D‐COS) analysis are performed from 25°C to 80°C. As shown in Figure 3e, with increasing temperature, the broad ν(O─H) band in the 3200–3600 cm^−1^ region exhibits a gradual decrease in absorbance intensity and evident band‐shape evolution, suggesting partial dissociation of O─H‐related hydrogen bonding associated with HEA segments. Considering the overlapped nature of the O─H stretching band, the components at 3370, 3478, and 3525 cm^−1^ are selected to describe different O─H microenvironments within the hydrogen‐bonding network. In the 1480–1800 cm^−1^ region, the spectra further show temperature‐dependent changes in C═O and [BMIM]^+^‐related vibrations. In the 1520–1800 cm^−1^ region, the component at 1755 cm^−1^ is assigned to ν(C═O)_free, while the band at 1726 cm^−1^ is attributed to ν(C═O)_interacted (Figure 3f). The weak feature around 1637 cm^−1^ is assigned to H‐bonded ν(C═O), and the band at 1570 cm^−1^ corresponds to ν(C═N). Upon heating, the temperature‐dependent change of ν(C═O)_free is relatively weak, suggesting that free or weakly interacting ester C═O groups are less sensitive to thermal perturbation. In contrast, the gradual decreases in the intensities of ν(C═O)_interacted and H‐bonded ν(C═O) indicate a progressive weakening of C═O‐involved hydrogen bonding and ion‐dipole interactions [48]. Meanwhile, the gradually decreased intensity of the ν(C═N) vibration of the imidazolium ring suggests a mild thermal perturbation and progressive weakening of the electrostatic interactions associated with [BMIM]^+^ [37, 49]. To further resolve the sequence of these thermally induced molecular events, 2D‐COS analysis is applied to the temperature‐dependent ATR‐FTIR datasets. According to Noda's rules [50] and the cross‐peak signs summarized in s S2 and S3, the synchronous and asynchronous spectra reveal a response sequence of 3370 → 3478 → 3525 cm^−1^ in the O─H region, corresponding to different O─H microenvironments within the hydrogen‐bonding network (Figure 3g(i,ii)). This sequence indicates that O─H‐related hydrogen bonding responds sequentially to thermal stimulation. Similarly, the 2D‐COS spectra in the 1520–1800 cm^−1^ region reveal a response sequence of 1755 → 1726 → 1570 → 1637 cm^−1^, corresponding to ν(C═O)_free → ν(C═O)_interacted → ν(C═N) → H‐bonded ν(C═O), respectively (Figure 3h(i,ii)). This sequence indicates that interaction‐affected ester C═O groups respond earlier to thermal stimulation than the electrostatic interactions associated with [BMIM]^+^ ν(C═N), suggesting that C═O‐involved hydrogen bonding and ion‐dipole interactions are relatively more sensitive to temperature variation. Overall, these results demonstrate that the thermally responsive O─H hydrogen‐bonding network, ester C═O microenvironment, and [BMIM]^+^‐related interactions jointly contribute to the structural stability and adaptive molecular rearrangement of the ionogel network.

In addition to thermal tolerance, the excellent adhesive property of the ionogel active layer is critical for its application in smart gloves, enabling direct integration with electrodes and ensuring accurate and stable sensing signal output. As shown in Figure S10, the P(BZMA‐co‐HEA)/IL ionogel exhibits favorable adhesion to a wide range of substrates, including copper, glass, paper, PDMS, plastic, PTFE, rubber, and wood. Lap‐shear tests are further conducted to quantify the adhesion strength on different substrates, with results presented in Figure 3i and Figure S11. The adhesion strengths on paper, copper, glass, and plastic reach 57.40, 53.64, 42.41, and 39.98 kPa, respectively. To evaluate adhesion durability, repeated adhesion tests are performed on each substrate (Figure 3j). The results show that although the adhesion strength slightly decreases after five cycles, even the substrate exhibiting the largest decline (plastic) retains approximately 77.21% of its initial adhesion strength. This favorable repeatable adhesion is attributed to dynamic non‐covalent interactions formed between the ionogel and the substrates (Figure S12). As summarized in Table S4, the as‐prepared P(BZMA‐co‐HEA)/IL ionogel exhibits better adhesion strength than several recently reported gel/ionogel materials on different substrates. More importantly, benefiting from excellent weight retention and environmental tolerance, the adhesive performance of the ionogel is further evaluated after 30 days of open exposure under both room temperature and 65°C conditions. As shown in Figure 3k, the ionogel maintains good adhesion strength to both glass and copper after prolonged exposure. Moreover, it also exhibits measurable adhesion strength underwater (Figure S13a). After adhesion to a plastic substrate and immersion in water for 24 h, no visible edge lifting or detachment is observed (Figure S13b). These results indicate that the ionogel possesses favorable adhesion stability and consistent environmental adaptability.

### 2.4 Anti‐Swelling Properties of Ionogel Active Layers

In conventional high‐water‐content polymer systems, material swelling in aqueous environments often leads to significant deterioration of mechanical properties, thereby limiting their practical applicability. Effective regulation of swelling behavior is therefore essential for maintaining the structural stability of ionogel active layers and ensuring reliable sensing performance in aquatic environments. The swelling behavior of PHEA gel in both water and seawater is first evaluated (Figure S14a,b), showing a remarkably high swelling ratio of 503.34% in water, indicating severe network expansion. This pronounced swelling originates from the strong hydrophilicity of PHEA chains, which facilitates continuous water penetration into the polymer network and ultimately disrupts structural integrity. To enhance structural stability, hydrophobic BZMA monomers and hydrophobic IL are introduced into the polymer system. Through a phase separation mechanism that promotes aggregation of hydrophobic chains, an ionogel active layer with excellent anti‐swelling capability is constructed. The effectiveness of this strategy is verified by comparing the swelling behaviors of PHEA/IL and P(BZMA‐co‐HEA)/IL ionogels. As shown in Figure 4a, a clear visual difference in swelling behavior is observed after immersion in water and seawater. The P(BZMA‐co‐HEA)/IL ionogel shows negligible dimensional change after 10 days of immersion, accompanied by a color transition from transparent to white, which is characteristic of phase separation. In contrast, the PHEA/IL ionogel exhibits significant swelling. Real‐time swelling ratio monitoring further supports this observation (Figure 4b). The experimental results confirm the excellent anti‐swelling properties of the P(BZMA‐co‐HEA)/IL ionogel, with swelling ratios of 17.24% in water and 2.94% in seawater, due to a reduction in the osmotic pressure difference [51]. In contrast, the PHEA/IL ionogel exhibits much higher swelling ratios of 340.63% in water and 209.03% in seawater after only one day of immersion. The influence of aqueous environments on mechanical properties is further evaluated through tensile testing. As shown in Figure 4c,d, the P(BZMA‐co‐HEA)/IL ionogel retains favorable mechanical properties and can still withstand large tensile deformation after 10 days of immersion in water and seawater. By contrast, although the PHEA/IL ionogel exhibits reduced swelling compared to pure PHEA gel, it suffers from severe deterioration in mechanical integrity, leading to pronounced brittleness (Figure S15). The water contact angle (WCA) measurements were further performed to verify the hydrophobic‐regulation mechanism of the ionogel. As shown in Figure 4e, the WCA increases from 27.9° to 79.2° after the introduction of BZMA, indicating that the incorporation of BZMA increases the content of hydrophobic functional groups and significantly enhances surface hydrophobicity. In addition, the WCA of the P(BZMA‐co‐HEA)/IL ionogel further increases after water immersion (Figure S16), suggesting the formation of a water‐induced hydrophobic barrier at the ionogel surface. Collectively, the hydrophobic regulation mainly contributed by BZMA‐rich segments effectively suppresses water penetration while preserving the mechanical integrity of the ionogel, thereby enabling stable performance under aqueous conditions.

**FIGURE 4:**
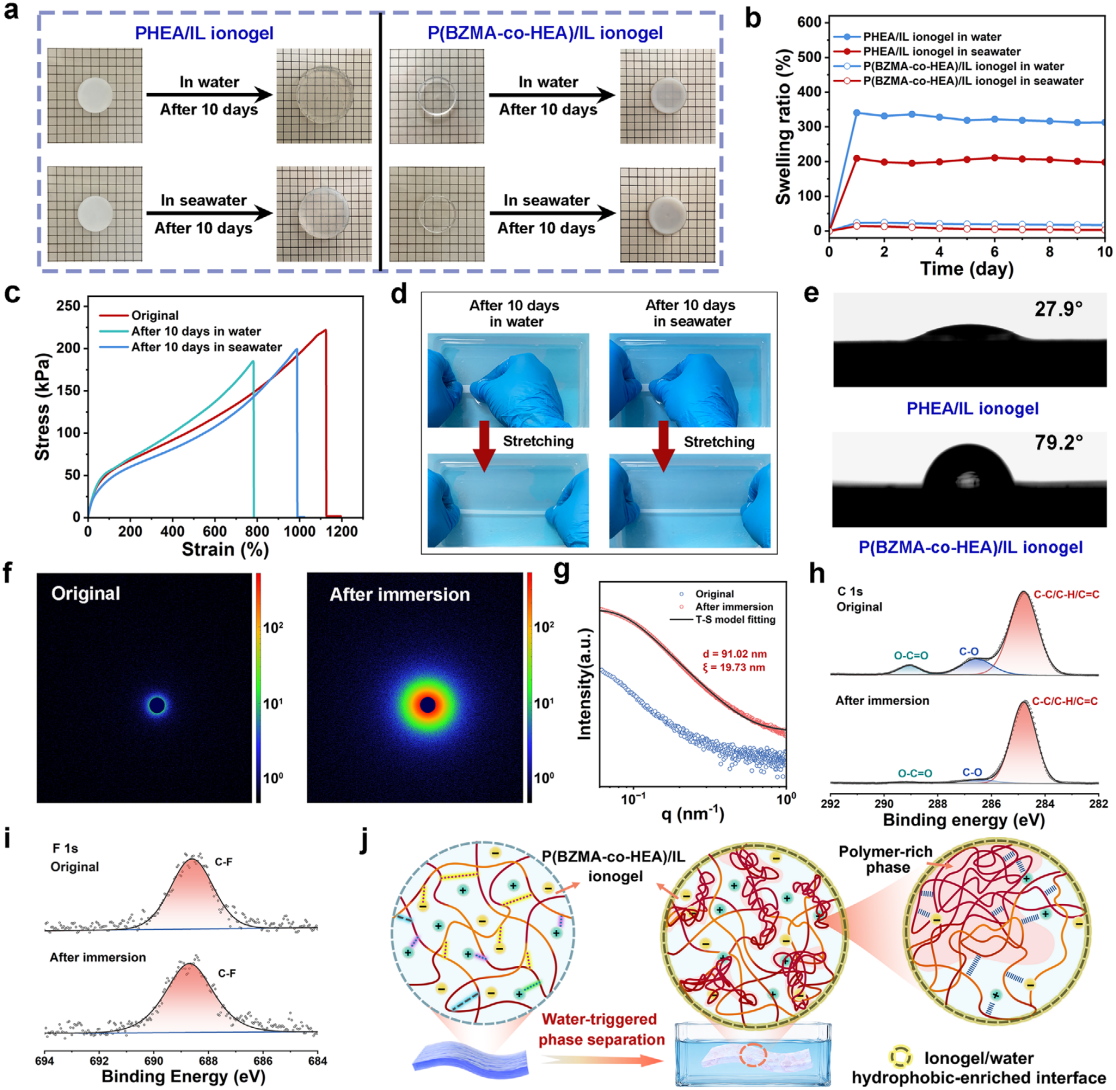
Anti‐swelling behavior and analysis of water‐induced microstructures in the P(BZMA‐co‐HEA)/IL ionogel. (a) Optical images of PHEA/IL and P(BZMA‐co‐HEA)/IL ionogels before and after immersion in water and seawater for 10 days. (b) Swelling ratio curves of PHEA/IL and P(BZMA‐co‐HEA)/IL ionogels in water and seawater over a period of 10 days. (c) Stress–strain curves and (d) tensile photographs of P(BZMA‐co‐HEA)/IL ionogel after 10 days of immersion in water and seawater. (e) WCAs on the surfaces of PHEA/IL and P(BZMA‐co‐HEA)/IL ionogels. (f) 2D SAXS patterns of P(BZMA‐co‐HEA)/IL ionogel before and after immersion. (g) 1D SAXS profiles and T‐S model. (h) C 1s and (i) F 1s XPS spectra of P(BZMA‐co‐HEA)/IL ionogel before and after immersion. (j) Schematic illustration of hydrophobic aggregation and water‐induced phase separation in P(BZMA‐co‐HEA)/IL ionogel before and after immersion.

To further probe the water‐triggered microstructural reconfiguration, SAXS, FTIR, and X‐ray photoelectron spectroscopy (XPS) analyses were conducted on the P(BZMA‐co‐HEA)/IL ionogel before and after immersion. SAXS analysis was performed to probe the water‐induced microstructural reconfiguration of the P(BZMA‐co‐HEA)/IL ionogel. As shown in Figure 4f, the pristine ionogel displays only a weak and diffuse scattering halo, indicating the absence of pronounced nanophase segregation within the probed length scale. After immersion, a distinct enhancement in scattering intensity is observed, suggesting the formation of water‐induced phase‐separated domains [52]. The corresponding 1D SAXS profile is fitted using the Teubner–Strey (T–S) model [53, 54], which gives a periodicity parameter d of 91.02 nm and a correlation length ξ of 19.73 nm for the immersed ionogel (Figure 4g). These parameters reflect the characteristic periodicity and size features of the chain‐aggregated regions within the ionogel. Quantitative Guinier analysis is further conducted in the low‐q region (*q* < 0.0100 Å^−1^) by fitting to the Guinier approximation, $\mathrm{In}I(q)=\mathrm{In}I(0)-\frac{q^{2}Rg^{2}}{3}$. The calculated radius of gyration Rg is 20.04 nm, corresponding to an estimated domain diameter of approximately 51.75 nm, further confirming the presence of nanoscale phase‐separated domains after immersion (Figure S17a) [55]. In addition, the local structural features are analyzed according to Porod's law, log[I(q)−B] = log(A)−n*log(q), where the Porod exponent n is extracted from the slope in the high‐q region (typically < 1 nm^−1^). As shown in Figure S17b, the immersed ionogel exhibits a Porod slope of 4.02, indicating the formation of phase‐separated domains with relatively smooth interfaces. In contrast, the original ionogel exhibits a Porod slope of 2.16, indicating that its local structure tends to resemble a Gaussian‐chain‐like configuration (Figure S17c) [54]. These SAXS results demonstrate that water immersion induces hydrophobic aggregation and nanoscale phase separation within the ionogel network. Moreover, FTIR spectra show that the ─OH stretching band shifts from 3525 to 3454 cm^−1^ and the C═O vibration slightly shifts from 1726 to 1723 cm^−1^ after immersion, indicating water‐induced reorganization of the hydrogen‐bonding environment within the ionogel network (Figure S18). A similar hydrophobic enrichment behavior is observed from the XPS results. After 10 days of water immersion, the characteristic signals in the survey spectrum, as well as the high‐resolution C 1s, O 1s, F 1s, and S 2p spectra, remain clearly detectable, indicating that the main functional groups and ionic components of the ionogel are preserved after prolonged exposure to water (Figure S19a). In the high‐resolution C 1s spectra, the C─C/C─H/C═C signal becomes more pronounced, accompanied by weakened oxygen‐containing C─O and O─C═O components (Figure 4h). Consistently, the O 1s spectra show a reduced C─OH signal (Figure S19b). This surface compositional shift, characterized by the preferential exposure of nonpolar C─C/C─H/C═C moieties including BZMA‐derived benzyl groups and the decreased exposure of hydrophilic oxygen‐containing groups, suggests hydrophobic‐interaction‐driven aggregation of polymer chains [53]. Meanwhile, the retained F 1s and S 2p signals associated with the TFSI^−^ anion demonstrate that the IL is not completely leached out but remains retained within the polymer network (Figure 4i and Figure S19c). Based on the WCA, SAXS, FTIR, and XPS results, a water‐induced phase separation mechanism is proposed (Figure 4j). In the air‐exposed pristine state, BZMA‐rich hydrophobic segments and ionic liquid domains are relatively dispersed within the polymer network. Upon water immersion, the incompatibility between hydrophobic segments and water promotes the aggregation and interfacial enrichment of BZMA‐rich chains. The resulting hydrophobic‐enriched interfacial layer suppresses further water penetration, while the ionic liquid remains retained within the polymer network, thereby contributing to the anti‐swelling stability of the ionogel.

### 2.5 Sensing Performance of P(BZMA‐co‐HEA)/IL Ionogel Sensor

As signal transmission in the smart glove relies on the strain‐induced resistance variation (ΔR/R_0_) of the ionogel active layer, the sensing performance of the P(BZMA‐co‐HEA)/IL ionogel is of central importance. For electrical characterization, ionogel sensors are fabricated by attaching electrodes composed of commercially available copper foil and copper wires to both ends of the ionogel (Figure 5a). The sensitivity of the sensor is quantified from the strain‐ΔR/R_0_ relationship. As shown in Figure 5b, the calculated gauge factors (GF) reach 1.52 in the strain range of 0%–150%, 2.57 at 150%–350%, and 3.22 at 350%–700%, demonstrating high sensitivity across an exceptionally wide strain range. The dynamic sensing performance is further evaluated through real‐time ΔR/R_0_ measurements under repeated loading–unloading cycles. As presented in Figure 5c,d, the sensor exhibits stable and distinguishable electrical responses under both small strains (10%–70%) and large strains (100%–700%), indicating reliable strain recognition capability over a broad deformation window. The rapid sensing response is further demonstrated by a random loading–unloading cycle, which reveals short response and recovery times of only 70 and 80 ms, respectively (Figure 5e). After 1000 stretch‐release cycles at 100% strain, the ionogel sensor exhibits negligible signal attenuation, demonstrating excellent cyclic stability (Figure S20). Moreover, the ionogel maintains stable ionic conductivity both after repeated stretching and under a 200 g external load, further highlighting its sensing durability and stability (Figure S21). Beyond ambient conditions, the ionogel demonstrates strong potential for underwater applications owing to its anti‐swelling characteristics and robust mechanical integrity. To evaluate its underwater sensing capability, sensing responses are recorded under various strain levels in aqueous environments. As shown in Figure S22a,b, the sensor clearly distinguishes strain‐dependent resistance signals, while maintaining stable signal output over 200 stretching cycles at 100% strain underwater (Figure S23). Quantitative ionic conductivity measurements show that after an initial decrease, the conductivity remains stable from 4 to 10 days (Figure S24a,b). These results confirm reliable sensing performance in aquatic environments, meeting the requirements for continuous and accurate hand motion monitoring during diving activities. In addition, after thermal aging at 65°C for 30 days, the sensor retains a high GF value of 2.78 (Figure S25) and stable cyclic performance (Figure S26a,b), demonstrating excellent thermal stability and long‐term service reliability.

**FIGURE 5:**
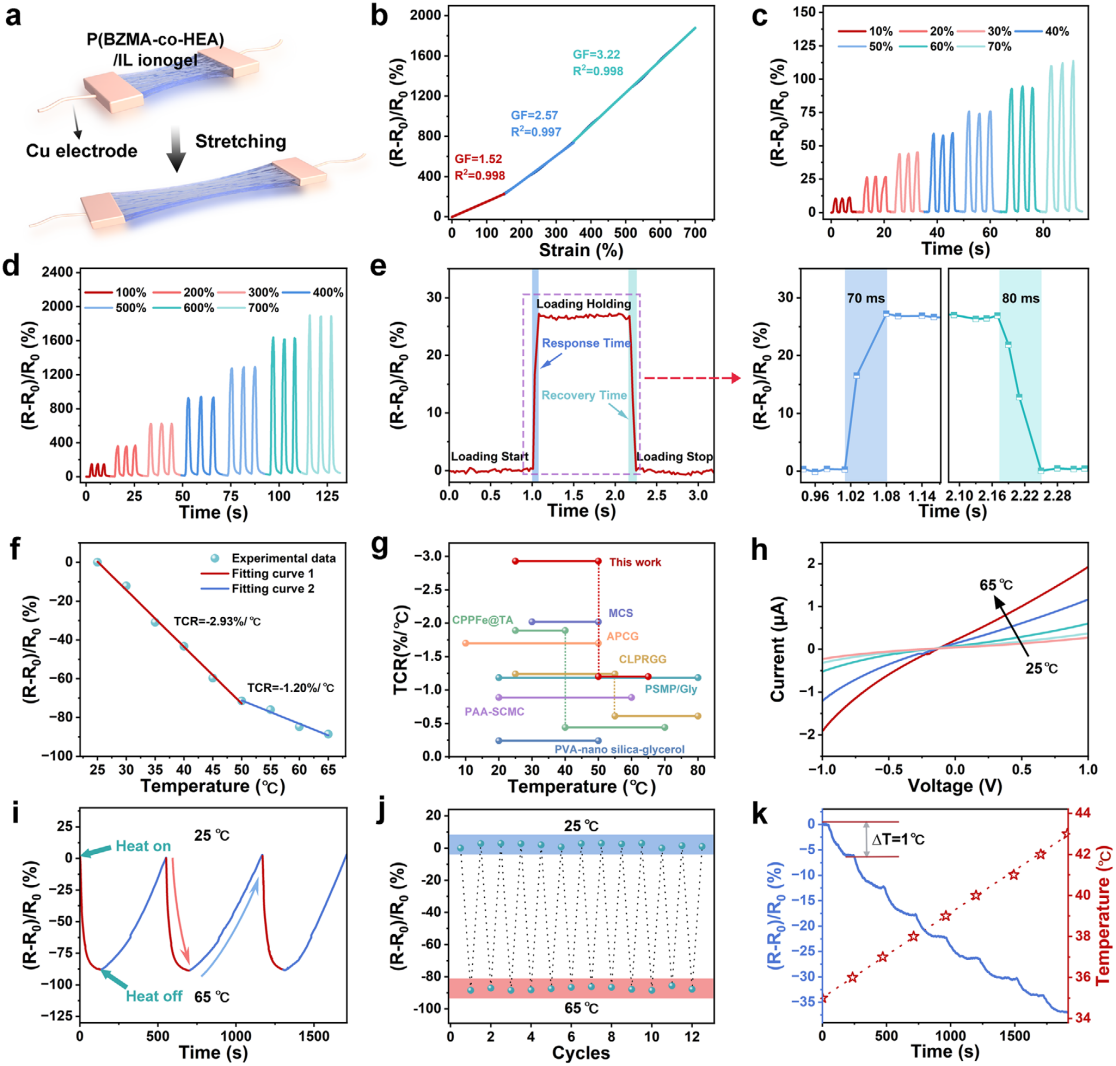
Sensing properties of the P(BZMA‐co‐HEA)/IL ionogel sensor. (a) Schematic diagram of the assembled ionogel‐based sensor. (b) ∆R/R_0_‐strain curve of the ionogel sensor. ∆R/R_0_ of the ionogel sensor under (c) 10%–70% strain and (d) 100%–700% strain. (e) Response/recovery time of the ionogel sensor at 20% strain. (f) ∆R/R_0_ of the ionogel sensor in the range of 25°C to 65°C. (g) Comparison of the TCR value with previously reported flexible temperature sensors. (h) Current–voltage (*I*–*V*) curves of the ionogel sensor from 25°C to 65°C. (i) On/off response of the ionogel sensor upon heating to 65°C. (j) Temperature sensing stability for 12 cycles between 25°C and 65°C. (k) ∆R/R_0_ in the temperature range of 35°C to 43°C at 1°C intervals.

Benefiting from the intrinsic temperature tolerance of the P(BZMA‐co‐HEA)/IL ionogel, its feasibility for temperature sensing is further investigated. The thermal response is evaluated using the temperature coefficient of resistance (TCR), defined as *TCR =* [(*R*_T_−*R*_0_)/*R*_0_]/Δ*T*, where *R*_T_ and *R*_0_ represent the resistance at a given temperature and the initial resistance, respectively, and ΔT denotes the temperature variation [56]. Within the temperature range of 25°C–65°C, the sensor exhibits a pronounced negative temperature coefficient, characterized by a linear decrease in resistance with increasing temperature (Figure 5f). The calculated TCR values reach −2.93%°C^−1^ from 25°C to 50°C and −1.20%°C^−1^ from 50°C to 65°C, outperforming most previously reported temperature sensors (Figure 5g and Table S5). Meanwhile, the ionic conductivity increases from 3.34 to 22.01 mS m^−1^ as the temperature rises from 25°C to 65°C, reaching approximately 6.6 times the room‐temperature value (Figure S27). *I*–*V* characterization further confirms this negative temperature coefficient behavior, with an increasing slope observed at elevated temperatures, indicating reduced resistance (Figure 5h). The sensor also exhibits rapid and stable switching behavior, as demonstrated by consistent on/off sensing responses over three cycles at 65°C (Figure 5i), suggesting excellent dynamic thermal responsiveness. Stable signal output is maintained over 12 repeated heating–cooling cycles across 25°C–65°C (Figure 5j), indicating high thermal reproducibility. Based on these reliable temperature response characteristics, we have developed a temperature alarm system. Its operational principle is illustrated in Figure S28, with additional details provided in Notes S1 and S2, Figure S29, and Video S1. The system continuously monitors the ambient temperature in real time and automatically triggers both visual and auditory alarms when the temperature exceeds a preset safety threshold, providing effective overheating warnings in high‐temperature environments. This system demonstrates the practical application of the P(BZMA‐co‐HEA)/IL ionogel sensor in temperature monitoring and safety warning scenarios. Furthermore, the sensor is capable of detecting subtle temperature variations, as verified by simulated human body temperature changes from 35°C to 43°C with 1°C increments (Figure 5k). This capability highlights its suitability for both wide‐range thermal monitoring and precise detection of minor temperature fluctuations, which is essential for tracking diver body temperature and preventing hypothermia.

### 2.6 Smart Glove System Integrating Underwater Communication and Safety Warning

With the increasing number of swimmers and divers participating in underwater exploration and activities, there is a growing demand for high‐performance underwater communication and motion sensing technologies [57, 58, 59]. Conventional underwater communication devices suffer from inherent drawbacks such as bulky structure, high cost, and limited flexibility, which restrict their practical use in rapid and intuitive information exchange during underwater operations. Wearable smart gloves integrated with flexible sensors have therefore attracted increasing attention as an emerging platform for underwater communication. By accurately recognizing hand gestures and mapping them to predefined commands, such devices enable efficient information transmission, thereby improving operational efficiency and safety for divers.

Benefiting from the stable sensing response of P(BZMA‐co‐HEA)/IL ionogel sensors in aqueous environments, a smart glove system for underwater communication and safety monitoring is developed. The system is capable of identifying 12 predefined gestures to convey different instructions, with the detailed gesture‐command relationship shown in Figure S30. During underwater operation, if Diver 2 wearing the smart glove encounters a potential hazard (e.g., a shark approaching), information can be rapidly transmitted through a “Danger” gesture. Upon receiving this signal, Diver 1 can respond with an “OK” gesture to confirm message reception, thereby effectively reducing potential risks. Furthermore, if Diver 3 experiences an emergency accompanied by abnormal or continuous hand bending, the system recognizes this behavior in real time and transmits an alert to the ground monitoring terminal, demonstrating its applicability in complex underwater environments (Figure 6a). The functional workflow of the smart glove system is illustrated in Figure 6b and mainly consists of three modules: signal acquisition, signal processing, and information display. Ten P(BZMA‐co‐HEA)/IL ionogel sensors are integrated into the glove and aligned with finger joints to collect sensing signals generated by finger motion in real time. These signals are converted into digital values through an analog‐to‐digital conversion (ADC) module and subsequently processed by the ESP32 microcontroller. The acquired data are then analyzed using a neural network algorithm to achieve accurate gesture recognition. For real‐time visualization, a Python‐based interface is developed using PyQt5. The sensing data are transmitted wirelessly from the ESP32 to the interface via Wi‐Fi, allowing simultaneous display of raw signals and gesture recognition results derived from neural network analysis. The final software interface is shown in Figure S31. As illustrated in Figure 6c, the ionogel sensor array consists of ten identical sensing units, labeled Ch1 to Ch10 according to their spatial positions and corresponding data acquisition channels. The assembled smart gloves are tested in both water and air by performing the 12 predefined gestures to verify their capability for continuous motion capture. As shown in Figure 6d and Figure S32, the sensing signals corresponding to different gestures are clearly distinguishable, demonstrating stable and accurate gesture recognition performance in both environments. To construct a reliable gesture‐recognition model, 1000 samples are collected for each gesture category, forming independent datasets for water and air environments. The datasets are randomly divided into training, validation, and test sets with a ratio of 4:1:1 for model training and performance evaluation. Figure 6e presents the visualization of the underwater gesture dataset distribution after dimensionality reduction using t‐distributed stochastic neighbor embedding (t‐SNE). Distinct clustering boundaries between gesture categories are observed, indicating strong intra‐class consistency and clear inter‐class separability. Based on these datasets, an FCNN model is constructed, consisting of an input layer, three hidden layers, and an output layer (model structure shown in Figure S33 and specific parameter configuration in Note S3). Figure 6f shows the evolution of model accuracy and loss during 100 training epochs for underwater gesture recognition. The trained model achieves an average recognition accuracy of 99.92% on the test set, with the confusion matrix presented in Figure 6g. All gesture categories except Gesture 1 reach 100% recognition accuracy. Meanwhile, the air‐environment gesture recognition model achieves 100% accuracy (Figure S34), demonstrating the robustness of the sensing system in different environments. To further validate practical applicability, the trained model is deployed onto a host computer for real‐time underwater gesture recognition. As shown in Figure 6h, when the diver sequentially switches gestures (i → ii → iii → iv), the software interface synchronously updates and displays the corresponding recognition results (Video S2). These results confirm that the smart glove system enables continuous and accurate gesture recognition in realistic underwater scenarios such as swimming and diving.

**FIGURE 6:**
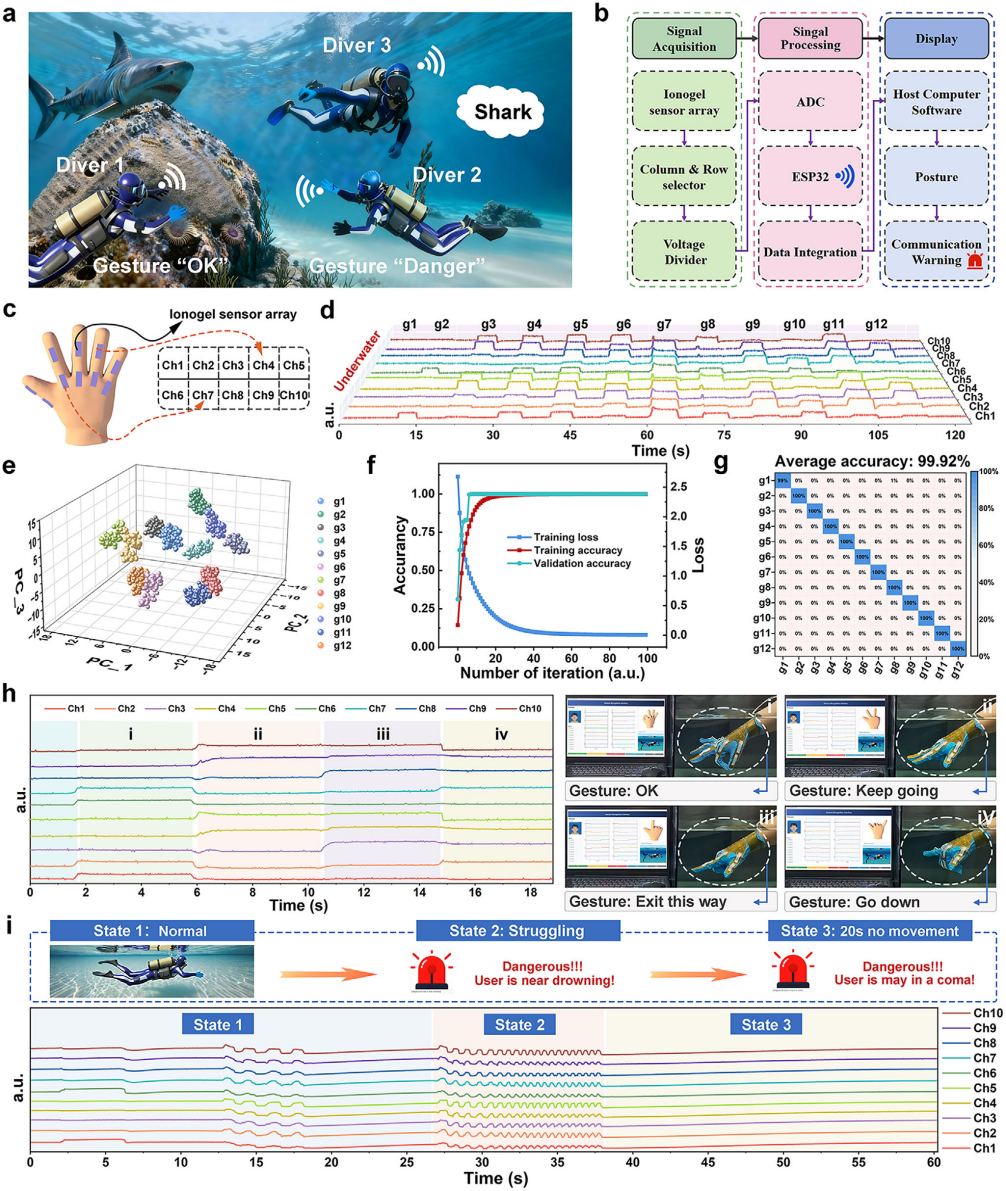
Smart glove system integrating underwater communication and safety warning. (a) Schematic of the system application scenario. (b) System workflow diagram. (c) Correspondence between the ionogel sensor array and signal channels (Ch1‐Ch10). (d) Response curves of the smart glove to continuous gesture changes underwater. (e) Visualization of the underwater gesture dataset distribution based on t‐SNE dimensionality reduction. (f) Variation curves of accuracy and loss for the FCNN‐based underwater gesture recognition model over 100 training epochs. (g) Confusion matrix of the trained FCNN model. (h) Demonstration of the system's underwater gesture recognition capability. (i) Demonstration of the system's long‐term status monitoring and safety warning function.

Furthermore, for extreme situations such as drowning or loss of consciousness, the system integrates a long‐term status monitoring function based on real‐time signal analysis using a sliding time window. When the standard deviation of the signals within the time window exceeds a preset threshold, a timer is activated. If this abnormal fluctuation persists for 10 s, the system identifies the state as a drowning struggle condition (State 2) and triggers an alarm displaying the message “Dangerous!!! User is near drowning!”. In addition, when the signal fluctuation remains consistently low within a 20 s time window, the system determines that the user may be in a coma (State 3) and immediately issues an alert message “Dangerous!!! User may be in a coma!”. As shown in Figure 6i, the system reliably distinguishes three diver states. State 1 corresponds to normal activity, during which the diver can communicate and move freely without triggering alarms. When State 2 or State 3 occurs, the system responds immediately and generates corresponding warning signals (Video S3). By enabling continuous movement state monitoring and rapid early warning, the smart glove system significantly enhances safety assurance during underwater activities. The real‐time feedback capability allows rescue teams to respond promptly, thereby reducing the risk of accidents in emergency situations. These results demonstrate the potential of the developed smart glove system for underwater communication and intelligent safety monitoring. The underwater gesture‐recognition test serves as a proof‐of‐concept demonstration under constant water‐temperature conditions. It should be noted that the ionogel exhibits a pronounced and linear temperature‐dependent resistance response over the range of 20°C–45°C (Figure S35), highlighting its potential for environmental temperature monitoring in addition to underwater strain sensing. In future studies, the integration of an independent ionogel‐based temperature reference unit will be further explored, together with adaptive signal‐decoupling algorithms, to enable the simultaneous acquisition and independent resolution of strain and temperature information.

## 3 Conclusions

In summary, this work develops a smart glove system based on a P(BZMA‐co‐HEA)/IL ionogel active layer. The ionogel is constructed through a network‐constrained ionic soft‐space regulation strategy, in which the copolymer network provides mechanical support and structural integrity, while the ionic soft spaces contribute to deformation compliance and stable ion transport. This molecular‐level regulation effectively balances mechanical reliability, environmental stability, and sensing adaptability, enabling the ionogel to maintain high stretchability, favorable adhesion, anti‐swelling capability, and stable sensing performance under different operating conditions. The performance comparison in Table S6 further highlights the balanced and comprehensive performance advantages of the ionogel. To translate the material advantages into a wearable system, the ionogel active layer is further integrated with a compact FPCB to construct a detachable and reconfigurable smart glove platform. This integrated design simplifies the conventional assembly process, improves structural flexibility, and ensures reliable sensor arrangement during hand motion. By combining multi‐channel sensing, FCNN‐assisted gesture recognition, and real‐time warning feedback, the smart glove realizes accurate gesture identification, underwater communication, and safety‐alert functions. Thus, this work provides a material‐design and device‐integration strategy for robust wearable sensing systems, showing potential for human motion monitoring, flexible interfaces, rehabilitation training, and hazardous‐environment operation.

## 4 Experimental Section

### 4.1 Materials

2‐Hydroxyethyl acrylate (HEA, 96%), benzyl methacrylate (BZMA, 98%), and 1‐Butyl‐3‐methylimidazolium bis(trifluoromethylsulfonyl)imide ([BMIM][TFSI], >98%) were provided by Shanghai Aladdin Biochemical Technology Co., Ltd. Ethylene glycol dimethacrylate (EGDMA) was purchased from Beijing Mreda Technology Co., Ltd. 2‐hydroxy‐2‐methylpropiophenone (Irgacure 1173, 97%) was purchased from Shanghai Macklin Biochemical Technology Co., Ltd. The PET double‐sided adhesive tape was purchased from Shenzhen Deyi Adhesive Tape Technology Co., Ltd. The PU membrane was obtained from Henan Shengjie Medical Devices Co., Ltd. All reagents were used without further purification.

### 4.2 P(BZMA‐co‐HEA)/IL Ionogel Active Layer Preparation

The P(BZMA‐co‐HEA)/IL ionogel active layer was synthesized by UV‐induced in situ copolymerization using BZMA and HEA as monomers, EGDMA as the crosslinker, Irgacure 1173 as the photoinitiator, and [BMIM][TFSI] as the ionic liquid medium. The preparation procedure was as follows: First, prescribed amounts of HEA (35, 50, and 60 wt.% relative to the total mass) and 0.8 g of BZMA were dissolved in [BMIM][TFSI] (22, 33, and 44 wt.% relative to the total mass) under continuous stirring at room temperature to form a homogeneous mixture. Subsequently, 1.5 µL of EGDMA and 20 µL of Irgacure 1173 were added, and the mixture was stirred until uniform. The resulting precursor solution was then poured into a polytetrafluoroethylene (PTFE) mold and cured under irradiation from a 60 W UV lamp (365 nm) for 3 min to obtain the ionogel. For comparative studies, control samples including PHEA gel, PHEA/IL iongel, and P(BZMA‐co‐HEA) gel were also prepared, where the HEA and [BMIM][TFSI] contents were fixed at 50 and 33 wt.%, respectively. The detailed formulations of all ionogel and control samples are summarized in Table S1.

### 4.3 Assembly of the Ionogel‐Based Smart Glove

First, the flexible printed circuit board (FPCB) was firmly attached to the glove substrate using PET double‐sided adhesive tape to establish a stable electronic platform. Ten segments of P(BZMA‐co‐HEA)/IL ionogel (10 mm × 35 mm × 1.5 mm) were then placed to bridge the corresponding electrode pairs at each joint sensing node on the FPCB, forming the core sensing units. To further enhance mechanical stability, a PU film was selectively applied to cover and secure the non‐joint portions of the ionogel, while the segments located at the joint bending areas remained exposed to the environment as the active sensing regions. This configuration ensures that the ionogel in the joint areas is directly in contact with water and functions as the primary sensing layer for mechanical deformation, while the PU‐covered sections provide structural fixation.

### 4.4 Characterization

The rheological properties were characterized using a rheometer (Mars 60, Haake) in dynamic oscillation mode. Viscosity measurements of the precursor solution and the cured ionogel were carried out over an angular‐frequency range of 0.1–100 Hz. Strain amplitude sweep from 0.1% to 100% at 25°C with an angular frequency of 10 rad s^−1^. Frequency sweep tests were conducted over the range of 0.1–10 Hz. Alternating strain tests were performed between 0.1% and 67% strain to evaluate the self‐recovery performance of the ionogel. Temperature‐dependent rheological behavior from 26.39°C to 79.48°C under 1% strain and 10 rad s^−1^ frequency. The chemical structures of HEA, BZMA, [BMIM][TFSI], P(BZMA‐co‐HEA) gel, and P(BZMA‐co‐HEA)/IL ionogel were analyzed using a Fourier transform infrared (FTIR) spectrometer (Nicolet iS5, Thermo Fisher). All spectra were recorded in the range of 4000–400 cm^−1^ at a spectral resolution of 4 cm^−1^. The transmittance spectrum of the P(BZMA‐co‐HEA)/IL ionogel was measured using a UV–vis spectrophotometer (TU‐1901, Puxi, China) with a wavelength range of 200–800 nm. Thermogravimetric analysis (TGA) was performed using a Mettler Toledo TGA/DSC 1/1600 instrument, heating the sample from 30°C to 600°C at 10°C min^−1^ in a N_2_ environment. The water contact angles (WCAs) were determined using a Dataphysics OCA25 contact angle meter. Dynamic mechanical analysis (DMA) was performed using a TA Instruments Q800 analyzer. The samples were tested over a temperature range from −50°C to 45°C at a heating rate of 5°C min^−1^. Small‐angle X‐ray scattering (SAXS) measurements were performed using a Xeuss 3.0 SAXS/WAXS system (Xenocs, France). Cu‐Kα radiation was used as the X‐ray source, with an X‐ray energy of 8.05 keV and a wavelength of λ = 1.54189 Å. The measurements were conducted under vacuum conditions (< 1 mbar), with an ionogel sample‐to‐detector distance of 1800 mm. Two‐dimensional SAXS patterns were recorded using an Eiger2R 1 M detector with a pixel size of 75 µm and were further azimuthally integrated to obtain one‐dimensional scattering intensity profiles. The scattering vector q was calculated according to q = (4π/λ)sinθ, where 2θ is the scattering angle. Variable‐temperature FTIR spectra were obtained on a Bruker Vertex 70 V FTIR spectrometer equipped with a Harrick temperature‐controlled in situ cell. X‐ray photoelectron spectroscopy (XPS) data were collected on a Thermo Scientific ESCALAB 250Xi+ spectrometer.

### 4.5 Mechanical Properties

The mechanical properties of the samples were determined using a universal testing machine (FBS‐100NS, China) at room temperature. The samples were prepared in a dumbbell shape with dimensions of 10 mm × 4 mm × 1.5 mm (length × width × thickness). Tensile tests were performed at a constant speed of 50 mm min^−1^. The elastic modulus was calculated as the linear region (ε = 10%–20%) of the stress–strain curve, while toughness was determined from the area under the curve. Each experiment was repeated at least three times.

### 4.6 Long‐Term Stability Tests

To evaluate the long‐term stability of ionogels under different environmental conditions, circular samples with a diameter of 2 cm were tested at room temperature (RT), elevated temperature (65°C), and underwater conditions (including both water and seawater). Specifically, the ionogels were continuously stored at RT and 65°C for 30 days, with their weights periodically measured and recorded. Additionally, the swelling behavior was investigated to assess their stability in aqueous environments. Prior to immersion testing, the initial weight (*W*_0_) of each sample was measured. The samples were then immersed in both water and simulated seawater (35 g L^−1^ natural sea‐salt solution) for 10 days. During this period, they were removed every 24 h, blotted dry with filter paper, and weighed (*W*_t_). The swelling ratio was calculated using the following formula: swelling ratio = (*W*_t_−*W*_0_)/*W*_0_ × 100%. Meanwhile, the mechanical properties of each sample after exposure to different environments were also investigated using a universal testing machine.

### 4.7 Adhesion Properties

The adhesion strength of ionogels on various substrates, including paper, copper sheets, glass, and plastic, was evaluated. The ionogel samples (20 × 20 mm^2^) were sandwiched between two untreated substrate surfaces and subjected to a 500 g weight for 20 min to ensure they fit completely. The adhesion performance was quantitatively measured using a universal testing machine (FBS‐100NS, China) through lap shear testing at a constant displacement rate of 50 mm min^−1^ at temperatures of 25°C and 65°C. Each type of substrate was tested at least three times to record the force–distance correlation curve. The adhesion strength was calculated as the maximum force normalized by the bonded area.

### 4.8 Conductivity Tests

Electrochemical impedance spectra (EIS) were recorded using an electrochemical workstation (CS350M, China) over a frequency range of 0.1–100 000 Hz with an AC amplitude of 10 mV under open‐circuit conditions. The ionogel samples were cut into rectangular specimens of 1 cm × 2 cm × 1 mm and sandwiched between two copper foils during the measurements. The ionic conductivity (σ) was determined by the equation σ = L/(RA), where L is the distance between the two copper foils, R is the electric resistance of the ionogels, and A is the contact area of the ionogels.

### 4.9 Sensing Properties

The electrical resistance variation of ionogels was tested using an LCR meter (TH2840B, China) and a semiconductor parameter analyzer (Keithley 2602B). The relative resistance change (∆*R*/*R*_0_) was determined by the equation (*R*−*R*_0_)/*R*_0_ × 100%, where R corresponds to the measured resistance under strain and *R*_0_ represents the original resistance. Subsequently, the strain sensitivity was evaluated by the gauge factor (GF), expressed as (∆*R*/*R*_0_)/ε, where ε represents the applied strain.

## Author Contributions

**Xiaoqin Li**: conceptualization, investigation, writing – original draft, writing – review and editing, validation, methodology, software, data curation, formal analysis, visualization. **Jianwen Liu**: conceptualization, investigation, writing – original draft, validation, visualization, writing – review and editing, software, formal analysis, data curation. **Wenhao Yang**: investigation, validation, visualization, software, formal analysis, writing – review and editing, methodology. **Wenjing Yue**: conceptualization, writing – review and editing, validation, software, methodology, supervision, investigation. **Namyoung Kim**: conceptualization, investigation, methodology, validation, writing – review and editing, software, formal analysis, supervision. **Young‐Kee Shin**: conceptualization, investigation, validation, writing – review and editing, formal analysis, supervision, methodology, visualization. **Song Gao**: conceptualization, investigation, methodology, visualization, writing – review and editing, formal analysis, supervision, writing – original draft. **Eun‐Seong Kim**: conceptualization, investigation, funding acquisition, writing – review and editing, writing – original draft, visualization, project administration, resources, methodology. **Yang Li**: conceptualization, writing – original draft, funding acquisition, visualization, writing – review and editing, project administration, supervision, resources, investigation, software.

## Conflicts of Interest

The authors declare no conflicts of interest.

## Supporting information




**Supporting File 1**: advs78325‐sup‐0001‐SuppMat.docx.

**Supporting File 2**: advs78325‐sup‐0002‐VideoS1.mp4.

**Supporting File 3**: advs78325‐sup‐0003‐VideoS2.mp4.

**Supporting File 4**: advs78325‐sup‐0004‐VideoS3.mp4.

## Data Availability

The data that support the findings of this study are available from the corresponding author upon reasonable request.

## References

[1] D. Yang , K. Zhao , R. Yang , et al., “A Rational Design of Bio‐Derived Disulfide CANs for Wearable Capacitive Pressure Sensor,” Advanced Materials 36, no. 30 (2024): 2403880, 10.1002/adma.202403880. 38723049

[2] K. Li , W. Yue , Y. Guo , Y. Li , G. Shen , and J. C. Lee , “Wearable Thumb Sleeves Enabled By Self‐Supervised Learning With Few Stretchable Sensors And Few‐Shot Data For Switchable Finger Tasks,” Science Advances 12 (2026): aea2020, 10.4028/b-4W7Te2. PMC13101860 42018630

[3] X. Chen , Z. Lou , X. Gao , et al., “A Noise‐Tolerant Human–Machine Interface Based On Deep Learning‐Enhanced Wearable Sensors,” Nature Sensors 1, no. 1 (2025): 39–51, 10.1038/s44460-025-00001-3.

[4] H. Li , H. Niu , F. Yin , W. Zhou , G. Shen , and Y. Li , “A Labor‐Division Cooperation Electronic Palm System for High‐Precision Crosstalk‐Free Cognition of Pressure and Temperature,” Advanced Materials 38, no. 8 (2026): 10241, 10.1002/adma.202510241. 41307294

[5] C. Wang , H. Niu , G. Shen , and Y. Li , “Self‐Healing Hydrogel‐Based Triboelectric Nanogenerator in Smart Glove System for Integrated Drone Safety Protection and Motion Control,” Advanced Functional Materials 35, no. 17 (2025): 2419809, 10.1002/adfm.202419809.

[6] M. Xiao , Y. Luo , H. Chen , et al., “Conformal Bioadhesive Hydrogel‐Based Multimodal Wristband for Accurate, Mobile, and Real‐Time Sign Language Translation and Human‐Machine Interactions,” Advanced Functional Materials 36, no. 19 (2026): 22153, 10.1002/adfm.202522153.

[7] Y. Zhou , S. Guo , Y. Zhou , et al., “Ionic Composite Nanofiber Membrane‐Based Ultra‐Sensitive and Anti‐Interference Flexible Pressure Sensors for Intelligent Sign Language Recognition,” Advanced Functional Materials 35, no. 29 (2025): 2425586, 10.1002/adfm.202425586.

[8] Y. Zhao , R. Wu , Y. Hao , et al., “Eco‐Friendly Multifunctional Hydrogel Sensors Enabled Sustainable and Accurate Human‐Machine Interaction System,” Advanced Materials 37, no. 32 (2025): 2507127, 10.1002/adma.202507127. 40395163

[9] Y. Luo , C. Liu , Y. J. Lee , et al., “Adaptive Tactile Interaction Transfer Via Digitally Embroidered Smart Gloves,” Nature Communications 15, no. 1 (2024): 868, 10.1038/s41467-024-45059-8. PMC10825181 38286796

[10] G. Xu , H. Wang , G. Zhao , et al., “Self‐Powered Electrotactile Textile Haptic Glove For Enhanced Human‐Machine Interface,” Science Advances 11, no. 12 (2025): adt0318, 10.1126/sciadv.adt0318. PMC11927614 40117358

[11] T. Wang , X. Xu , S. Li , et al., “Stretchable Curvature Sensors for Motion Capture With Bending‐Stretching Coupling Deformation,” Advanced Science 13, no. 12 (2026): 14779, 10.1002/advs.202514779. PMC12948215 41400093

[12] Y. G. Liang , W. H. Ran , D. Kuang , and Z. R. Wang , “Design Strategies And Insights Of Flexible Infrared Optoelectronic Sensors,” Journal of Semiconductors 46, no. 1 (2025): 011602, 10.1088/1674-4926/24080044.

[13] T. W. Kang , Y. J. Lee , B. Rigo , et al., “Soft Nanomembrane Sensor‐Enabled Wearable Multimodal Sensing and Feedback System for Upper‐Limb Sensory Impairment Assistance,” ACS Nano 19, no. 5 (2025): 5613–5628, 10.1021/acsnano.4c15530. PMC11823636 39888714

[14] X. Zhao , M. Yang , F. Li , et al., “A “Rigid‐Flexible” Iontronic Pressure Sensor With High Sensitivity And Wide Response Range For Hand Dysfunction Rehabilitation Training,” Chemical Engineering Journal 507 (2025): 160539, 10.1016/j.cej.2025.160539.

[15] S. Wen , P. Xia , C. Peng , et al., “Ultrahigh‐Water‐Content yet Robust Hydrogels Enabled by Bioinspired Laminated Membranous Network,” Advanced Materials 38, no. 4 (2026): 11595, 10.1002/adma.202511595. 41078002

[16] Y. Sun , G. Tian , W. Deng , and W. Yang , “Ionic Hydrogel Sensors Toward Next‐Gen Personalized Healthcare,” Advanced Materials 38, no. 3 (2026): 09122, 10.1002/adma.202509122. 41147149

[17] X. Jin , L. Song , M. Li , et al., “Non‐Drying Hydrogel Polyelectrolytes via Polymer‐Assisted Cation Solvation‐Confinement Toward H_2_O for Ultrahigh‐Voltage Aqueous Micro‐Supercapacitors,” Advanced Materials 37, no. 41 (2025): 09050, 10.1002/adma.202509050. 40702834

[18] Q. Ding , Y. Luo , W. Shi , et al., “Hydrogel‐Based Self‐Powered, Oxygen‐Resistant, and Flexible Sensors for Ultrasensitive and Selective NO_2_ Detection,” Advanced Functional Materials 36, no. 2 (2026): 12817, 10.1002/adfm.202512817.

[19] Y. Lin , J. Huang , S. Wang , et al., “Stretchable, Adhesive, Anti‐Freezing Hydrogel Electrolytes With Dual‐Functional Water Regulation Enabled by Amide Group–Salt–Water Interactions for All‐Climate Zinc‐Ion Batteries,” Advanced Materials 37, no. 41 (2025): 09975, 10.1002/adma.202509975. 40785419

[20] M. Gong , X. Wang , Z. Wu , et al., “Nature‐Inspired Molecular‐Crowding Enabling Wide‐Humidity Range Applicable, Anti‐Freezing, and Robust Zwitterionic Hydrogels for On‐Skin Electronics,” Small 20, no. 30, (2024): 2400161, 10.1002/smll.202400161. 38431936

[21] M. Zhang , Y. Yang , M. Li , et al., “Toughening Double‐Network Hydrogels by Polyelectrolytes,” Advanced Materials 35, no. 26 (2023): 2301551, 10.1002/adma.202301551. 36940146

[22] W. Gao , Z. Lu , S. Feng , et al., “3D Printable Dual‐Sensing Hydrogels With Exceptional Anti‐Swelling And Toughness,” International Journal of Extreme Manufacturing 8, no. 2 (2026): 025002, 10.1088/2631-7990/ae1c7d.

[23] L. Jiang , F. Li , Y. Li , et al., “Solvent‐Free Fabrication of Robust Physical Hydrogels via Bulk Copolymerization for Underwater Acoustics,” Advanced Materials 37, no. 38 (2025): 2508162, 10.1002/adma.202508162. 40538263

[24] S. Wang , H. Liu , Z. Yu , et al., “Cellulose‐Mediated Ionic Liquid Crystallization Enables Tough‐Stiff Switchable Ionogels,” Nature Communications 16, no. 1 (2025): 9007, 10.1038/s41467-025-64061-2. PMC12511293 41068099

[25] Z. Hu , Z. Han , H. Liu , et al., “Mechanically Strong and Tough Ionic Liquid Gel Electrolyte for Four‐Electron Zinc–Iodine Batteries,” Journal of the American Chemical Society 147, no. 50 (2025): 46632–46641, 10.1021/jacs.5c18431. 41329854

[26] A. Xu , J. Liu , Y. Wang , Z. Xiao , and Y. Xie , “Leaf‐Inspired Cellulose Ionogels Under Spatial Confinement With Synergistically Enhanced Mechanical and Sensing Performance for Flexible Sensors,” Advanced Functional Materials 36, no. 14 (2026): 19010, 10.1002/adfm.202519010.

[27] G. Du , J. Li , C. Wang , et al., “Tough Asymmetric Thermochromic Ionogels Via Dynamic In Situ Phase Separation For Dual‐Modal Smart Optical Switching,” Nature Communications 17 (2026): 4124. 10.1038/s41467-026-70830-4 PMC13150026 41844627

[28] H. Chen , C. Huang , K. Lu , Y. Gao , J. Yan , and Z. Wang , “Confinement‐Locking Strategy Enables Ionogels With Remarkable Mechanical Robustness and Fatigue Resistance,” Advanced Materials 38, no. 10 (2026): 20126, 10.1002/adma.202520126. 41451567

[29] Y. Xu , X. Jing , P. Feng , et al., “Breathable, Nanonet‐Reinforced Ultrathin Ionogel Film via Hydrogen Bonding‐Ion Dipole Synergy for Multifunctional Wearable Sensors,” Advanced Functional Materials 36, no. 10 (2026): 17882, 10.1002/adfm.202517882.

[30] X. Wang , S. Qin , J. Zhang , et al., “Multifunctional Elastic Supramolecular Hydrogels With Self‐Healing, Adhesive, and Ionic Thermoelectric Property for Flexible Sensing and Energy Harvesting,” Advanced Materials 38, no. 33 (2026): 73341, 10.1002/adma.73341. 42105198

[31] Y. Wang , S. Li , J. Han , et al., “Supramolecular Coupling Effect Enhanced Highly Transparent, Conductive Ionic Skin for Underwater Sensory and Interactive Robotics,” Advanced Materials 38, no. 9 (2026): 18076, 10.1002/adma.202518076. 41387941

[32] H. Zhai , W. Qin , Y. Wang , et al., ““Ionic Ocean” Constructed Super Underwater Adhesive, Stretchable, Self‐Healing Hydrophobic Conductive Ionogel,” Advanced Functional Materials 35, no. 41 (2025): 2505171, 10.1002/adfm.202505171.

[33] X. Tang , J. Liu , R. Tian , et al., “Microphase Separation‐Induced Conductivity Enhancement In Anti‐Swelling Amphiphilic Ionogels For Multimodal Sensing,” Chemical Engineering Journal 529 (2026): 172746, 10.1016/j.cej.2026.172746.

[34] X. Lyu , H. Zhang , S. Shen , Y. Gong , P. Zhou , and Z. Zou , “Multi‐Modal Sensing Ionogels With Tunable Mechanical Properties and Environmental Stability for Aquatic and Atmospheric Environments,” Advanced Materials 36, no. 45 (2024): 2410572, 10.1002/adma.202410572. 39292213

[35] J. Liu , X. Yang , M. Xu , et al., “Highly Stretchable, Self‐Adhesive, Ambient‐Stable, And Wide‐Temperature Adaptable Hydrophobic Ionogels For Wearable Strain Sensors,” Journal of Materials Chemistry C 11, no. 3 (2023): 1184–1196, 10.1039/D2TC04411K.

[36] Z. Yang , Y. Zhang , G. Wu , et al., “Internal Capsulation Via Self‐Cross‐linking and π‐Effects Achieves Highly Stable Perovskite Solar Cells,” Advanced Materials 36, no. 49 (2024): 2410425, 10.1002/adma.202410425. 39401268

[37] H. Huang , L. Sun , Y. Zhang , R. Zhang , C. Chen , and W. Sun , “Ionogels Reinforced by Ionophobic Coordination,” Advanced Materials 37, no. 34 (2025): 2506563, 10.1002/adma.202506563. 40504699

[38] J. Xu , H. Wang , X. Wen , S. Wang , and H. Wang , “Mechanically Strong, Wet Adhesive, and Self‐Healing Polyurethane Ionogel Enhanced With a Semi‐interpenetrating Network for Underwater Motion Detection,” ACS Applied Materials & Interfaces 14, no. 48 (2022): 54203–54214, 10.1021/acsami.2c15058. 36409304

[39] P. Shi , Y. Wang , W. W. Tjiu , C. Zhang , and T. Liu , “Highly Stretchable, Fast Self‐Healing, and Waterproof Fluorinated Copolymer Ionogels With Selectively Enriched Ionic Liquids for Human‐Motion Detection,” ACS Applied Materials & Interfaces 13, no. 41 (2021): 49358–49368, 10.1021/acsami.1c16081. 34632775

[40] X. Zhang , G. Zhang , X. Huang , J. He , Y. Bai , and L. Zhang , “Antifreezing and Nondrying Sensors of Ionic Hydrogels With a Double‐Layer Structure for Highly Sensitive Motion Monitoring,” ACS Applied Materials & Interfaces 14, no. 26 (2022): 30256–30267, 10.1021/acsami.2c08589. 35749282

[41] Q. Li , W. Li , Z. Liu , et al., “Poly(Ionic Liquid) Double‐Network Elastomers With High‐Impact Resistance Enhanced by Cation‐π Interactions,” Advanced Materials 36, no. 13 (2024): 2311214, 10.1002/adma.202311214. 38150638

[42] D. Lv , X. Li , X. Huang , et al., “Microphase‐Separated Elastic and Ultrastretchable Ionogel for Reliable Ionic Skin With Multimodal Sensation,” Advanced Materials 36, no. 17 (2024): 2309821, 10.1002/adma.202309821. 37993105

[43] S. Xu , S. Wu , R. Zhu , Z. Qiu , and Y. Yan , “Fully Physically Crosslinked PNIPAM Ionogels With High Mechanical Properties and Temperature‐Managed Adhesion Achieved by H_2_O/Ionic Liquid Binary Solvents,” Advanced Functional Materials 34, no. 41 (2024): 2405965, 10.1002/adfm.202405965.

[44] Z. Zhang , L. Qian , J. Cheng , C. Ma , and G. Zhang , “Neural Network‐Inspired Polyurea Ionogel With Mechanical Robustness, Low Hysteresis, and High Transparency for Soft Iontronics,” Advanced Functional Materials 34, no. 37 (2024): 2402115, 10.1002/adfm.202402115.

[45] M. Wang , X. Xiao , S. Siddika , et al., “Glassy Gels Toughened By Solvent,” Nature 631, no. 8020, (2024): 313–318, 10.1038/s41586-024-07564-0. 38898283

[46] X. Li , K. Cui , T. Kurokawa , et al., “Effect of Mesoscale Phase Contrast On Fatigue‐Delaying Behavior Of Self‐Healing Hydrogels,” Science Advances 7 (2021): abe8210. 10.1126/sciadv.abe8210 PMC8046377 33853776

[47] S. Wang , Z. Yu , X. Sun , et al., “A Universal Strategy to Mitigate Microphase Separation via Cellulose Nanocrystal Hydration in Fabricating Strong, Tough, and Fatigue‐Resistant Hydrogels,” Advanced Materials 37, no. 7 (2025): 2416916, 10.1002/adma.202416916. PMC11837898 39969391

[48] J. Zhang , W. Wang , Y. Zhang , et al., “Small‐Molecule Ionic Liquid‐Based Adhesive With Strong Room‐Temperature Adhesion Promoted By Electrostatic Interaction,” Nature Communications 13, no. 1 (2022): 5214, 10.1038/s41467-022-32997-4. PMC9445047 36064871

[49] Y. Ding , J. Xie , L. An , C. Yan , and F. Yan , “Covalent Adaptable Ionic Networks for Robust, On‐Demand Dismantlable and Fully Recyclable Adhesives,” Advanced Materials 38, no. 32 (2026): 73259, 10.1002/adma.73259. 42059597

[50] I. Noda , “Generalized Two‐Dimensional Correlation Method Applicable to Infrared, Raman, and other Types of Spectroscopy,” Applied Spectroscopy 47, no. 9 (1993): 1329–1336, 10.1366/0003702934067694.

[51] H. Wang , X. Lin , X. Li , et al., “Antiswelling, Ultrastretchable, and Ultrastable Hydrogel Sensors for Long‐Term Underwater Monitoring,” Small 21, no. 27 (2025): 2503067, 10.1002/smll.202503067. 40400497

[52] K. Cui , Y. N. Ye , T. L. Sun , et al., “Effect of Structure Heterogeneity on Mechanical Performance of Physical Polyampholytes Hydrogels,” Macromolecules 52, no. 19 (2019): 7369–7378, 10.1021/acs.macromol.9b01676.

[53] L. Han , C. Shan , M. Cui , R. Su , and R. Huang , “Ionic Liquid‐Mediated Phase Separated Hydrogels With Bicontinous Conductive Networks for Long‐Term Underwater Strain Sensing,” Small 21, no. 45 (2025): 06902, 10.1002/smll.202506902. 40988629

[54] L. Li , X. Wang , X. You , et al., “Super Stretchable Gelatin/Poly (Ionic Liquid) Hydrogel Enabled By Weak Hydrogen Bonds And Microphase Separation Towards Multifunctional And Self‐Powered Sensors,” Nano Energy 138 (2025): 110875, 10.1016/j.nanoen.2025.110875.

[55] L. Ren , G. Ma , Z. Wang , et al., “Ion‐Triggered Reconfigurable Hydrogels With Salt‐Enhanced Mechanical And Swelling Properties Via Network Topological Adaptation,” Nature Communications 17, no. 1 (2026): 4773, 10.1038/s41467-026-73723-8. PMC13219459 42191704

[56] T. Chen , J. Xu , C. Wang , et al., “Shape‐Memory Polyurethanes For Polar Wearables With Ultrasensitive Multi‐Monitoring,” Nature Communications 16, no. 1 (2025): 11329, 10.1038/s41467-025-66422-3. PMC12722725 41381552

[57] Y. Bai , Y. Shi , X. Li , Y. Zhang , and Y. Wang , “Cation‐π Interactions Based Conductive Hydrogels With Slide‐Ring Structure Toward Super Long‐Time in‐air/Underwater Linear Sensing and Communication,” Small 20, no. 50, (2024): 2406902, 10.1002/smll.202406902. 39363783

[58] Y. Q. Yang , Y. Li , D. Chen , and G. Z. Shen , “Advances in Flexible Weak‐Light Detectors Based On Perovskites: Preparation, Optimization, And Application,” Journal of Semiconductors 46, no. 1 (2025): 011608, 10.1088/1674-4926/24090046.

[59] L. Li , X. Fan , G. Chen , et al., “From Fish To Fiber: 3D‐Nanoprinted Optical Neuromast For Multi‐Integrated Underwater Detection,” Nature Communications 16, no. 1 (2025): 7390, 10.1038/s41467-025-62559-3. PMC12340067 40790294

