# Supplementary material for: An Environmental Stable Ionogel‐Based Smart Glove System for Integrated Underwater Communication and Safety Warning
Source: Adv Sci (Weinh). 2026 Oct 28:e78325. Online ahead of print. doi: 10.1002/advs.78325 (PMC13715916; doi:10.1002/advs.78325)
Supplement: Supplementary file 1 [file ADVS-9999-e78325-s002.docx]

**Supporting Information**

**An Environmental Stable Ionogel-Based Smart Glove System for Integrated Underwater Communication and Safety Warning**

*Xiaoqin Li, Jianwen Liu, Wenhao Yang, Wenjing Yue, Namyoung Kim, Young-Kee Shin, Song Gao,* Eun-Seong Kim,* and Yang Li**

**Note 1: The workflow diagram of the entire temperature alarm system：**

The workflow of the entire system can be divided into three parts (Figure S28). The first part is the signal acquisition circuit. Initially, a temperature change causes a variation in the sensor’s resistance. This variation is converted into an analog voltage signal by a voltage divider circuit, which is then captured and digitized by an Analog-to-Digital Converter (ADC). The second part is the signal processing circuit. The acquired signals are processed by the ESP32 microcontroller to formulate a data record of the sensor signals. The third part is the display and response module. The system analyzes the collected data, and once the temperature exceeds a preset high-temperature threshold, it triggers an alarm and response. On one hand, a high-temperature alarm message is displayed on the app interface. On the other hand, the system drives a SIM800C communication module to automatically place a call to a preset emergency contact, while simultaneously activating an alarm to sound an alert. This system achieves dual-alarm functionality through both sound/light prompts and remote notifications, ensuring timely and effective safety warnings.

**Note 2: The real-time temperature monitoring and alarm system based on the P(BZMA-co-HEA)/IL ionogel sensor：**

As shown in Figure S29, the ionogel sensor is placed on a hot plate, and the temperature is gradually increased from 25 °C to 65 °C to simulate environmental changes from RT to elevated temperature conditions. Throughout this process, the app receives and displays the temperature data in real-time. Once the monitored temperature exceeds the safety threshold of 60 °C, the system identifies a dangerous state. The app interface then automatically switches the status from “Normal” to “Warn”, and the alert icon changes from gray to red. Simultaneously, the system automatically places a call to pre-designated emergency contacts and triggers an on-site alarm to produce an audible alert, forming a complete remote and local alarm response. This system is suitable for high-temperature workplaces and residential environments, effectively preventing safety incidents such as fires.

**Note 3: Construction of FCNN:**

This FCNN is constructed using the PyTorch framework and adopts a five-layer architecture: an input layer with 10 nodes, three hidden layers (with 64, 32, and 16 neurons, respectively), and an output layer with 12 nodes. LeakyReLU activation functions and Dropout layers with a rate of 0.1 are incorporated between all layers to enhance nonlinear representation capacity and mitigate overfitting, thereby improving model robustness. During the training phase, the model undergoes 100 epochs of iteration. The cross-entropy loss function is employed to compute the error between predictions and ground-truth labels, while the gradient descent algorithm is applied to update the network weights.

**Figure S1.** a) Photographs of the front and reverse sides of the FPCB integrated within the smart glove. b) Schematic illustration depicting the integration of the ionogel active layer with the device, forming a conformal interface with the subject’s hand.

**Figure S2.** Frequency-dependent viscosity curves of the ionogel system before and after UV irradiation.

**Figure S3.** a) Stress and strain of ionogels with different HEA contents. (b) Stress and strain of ionogels with different IL contents.

**Figure S4.** The optical photographs of excellent mechanical properties for the P(BZMA-co-HEA)/IL ionogel, including a) stepwise stretching from 0% to 1100% and b) lifting a weight of at least 700 g.

**Figure S5.** a) Frequency sweep curves and b) step-strain time sweep curves under alternating strain for the P(BZMA-co-HEA)/IL ionogel.

**Figure S6. Full DMA curves and derivative analysis of ionogels with different HEA and IL contents.** a) Temperature-dependent DMA curves of P(BZMA-co-HEA)/IL ionogels with different compositions, including storage modulus (E'), loss modulus (E''), and tanδ. The glass transition temperature (Tg) is determined from the tanδ peak. (b) Derivative curves of d(log_10_ E')/dT for the corresponding ionogels. The minimum of each derivative curve represents the temperature at which (E') exhibits the maximum drop rate, reflecting the most pronounced modulus-softening process during heating. The shaded regions highlight the main modulus-drop regions. It should be noted that the derivative-derived temperatures are used to assist the analysis of network softening behavior and are not equivalent to (Tg), which was determined from the tanδ peak.

**Figure S7.** 2D SAXS patterns of 60% HEA-33% IL, 50% HEA-33% IL, and 50% HEA-44% IL ionogels.

**Figure S8.** I(q)*q^2^-q **plot of the** 50% HEA-44% IL ionogel**.** The low-q broad shoulder is located at approximately q = 0.0070 Å^−1^. The corresponding apparent characteristic length is calculated using L = 2π/q, giving L ≈ 89.7 nm. This length represents the apparent correlation scale of local compositional fluctuations or ionic soft spaces rather than the exact domain diameter.

**Figure S9.** Optical photographs of the P(BZMA-co-HEA)/IL ionogels being stretched after 30 days of storage at RT and 65 ℃.

**Figure S10.** Optical photographs of the P(BZMA-co-HEA)/IL ionogel adhering to different substrates.

**Figure S11.** The P(BZMA-co-HEA)/IL ionogel adhesion strength on various surfaces.

**Figure S12.** Adhesion mechanism of P(BZMA-co-HEA)/IL ionogel.

**Figure S13.** a) Underwater adhesion strength of the P(BZMA-co-HEA)/IL ionogel on glass and copper substrates. b) Optical images of the ionogel attached to a plastic substrate before and after immersion in water for 24 h.

**Figure S14.** a) Optical photographs of PHEA gels before and after immersion in water and seawater for 10 days. b) Swelling ratio curves of PHEA gels in water and seawater over a period of 10 days.

**Figure S15.** Stress-strain curves of PHEA/IL ionogel after 10 days of immersion in water and seawater.

**Figure S16.** WCAs on the surface of P(BZMA-co-HEA)/IL ionogel after 0, 5, and 10 days of immersion in water.

**Figure S17.** Guinier and Porod analyses of the P(BZMA-co-HEA)/IL ionogel. a) Guinier fitting of the immersed ionogel in the low-q region. b,c) Porod plots of the ionogel after and before immersion, respectively.

**Figure S18.** FTIR spectra of the P(BZMA-co-HEA)/IL ionogel before and after water immersion, showing the changes in the –OH stretching band and C=O stretching vibration.

**Figure S19.** a) XPS survey spectra, b) O 1s and c) S 2p XPS spectra of the P(BZMA-co-HEA)/IL ionogel before and after immersion.

**Figure S20.** Long-term stabilities of the P(BZMA-co-HEA)/IL ionogel sensor during 1000 cycles at 100% strain.

**Figure S21.** a) EIS curves and b) corresponding ionic conductivity in the original state, after 1000 cycles, and under a 200 g load.

**Figure S22.** ∆R/R_0_ of the P(BZMA-co-HEA)/IL ionogel sensor under a) 50%-250% strain and b) 300%-700% strain in the water.

**Figure S23.** Cycling stability of the P(BZMA-co-HEA)/IL ionogel sensor at 100% strain in water media.

**Figure S24.** a) EIS curves and b) corresponding ionic conductivity of the ionogel after soaking for 1-10 days.

**Figure S25.** The GF values of the P(BZMA-co-HEA)/IL ionogel after 30 days at 65°C.The values reach 1.43 (0-150%), 2.43 (150-350%), and 2.78 (350-700%), demonstrating that the ionogel sensor maintains high sensitivity after storage at 65 ℃ for 30 days.

**Figure S26.** Load-unload curves of the P(BZMA-co-HEA)/IL ionogel after storage at 65 ℃ for 30 days: (a) at 100%-700% strain and (b) cyclic at 100% strain.

**Figure S27.** a) EIS curves and b) corresponding ionic conductivity of the ionogel at different temperatures from 25 to 65 ℃.

**Figure S28.** Workflow diagram of the temperature alarm system.

**Figure S29.** The real-time temperature monitoring and alarm system based on the P(BZMA-co-HEA)/IL ionogel sensor.

**Figure S30.** Command mappings of 12 hand gestures in water and air environments.

**Figure S31.** PyQt5-based python software interface.

**Figure S32.** Response curves of the smart glove to continuous gesture changes in air.

**Figure S33.** FCNN-based flowchart for data acquisition and gesture recognition.

**Figure S34. Confusion matrix of the FCNN model for air gesture recognition.**

**Figure S35.** Underwater temperature response of the ionogel, showing the relative resistance change under different water temperatures from 20 to 45 ℃.

**Table S1. Detailed formulations of P(BZMA-co-HEA)/IL ionogels.**

| **Sample** | **HEA (g)** | **BZMA (g)** | **IL (g)** | **EGDMA (μL)** | **1173 (μL)** |
| --- | --- | --- | --- | --- | --- |
| **35% HEA** | **1.24** | **0.8** | **1.5** | **1.5** | **20** |
| **50% HEA** | **2.3** | **0.8** | **1.5** | **1.5** | **20** |
| **60% HEA** | **3.45** | **0.8** | **1.5** | **1.5** | **20** |
| **22% IL** | **2.3** | **0.8** | **0.86** | **1.5** | **20** |
| **33% IL** | **2.3** | **0.8** | **1.5** | **1.5** | **20** |
| **44% IL** | **2.3** | **0.8** | **2.43** | **1.5** | **20** |

**Table S2. Signs of the main cross-peaks in 2DCOS synchronous and asynchronous spectra from 3600-3200 cm^-1^.**

| **Cross-peak signs (left: synchronous; right: asynchronous)** | | | |
| --- | --- | --- | --- |
| **3370** | **-, -** | **+, -** |  |
| **3478** | **-, +** |  |  |
| **3525** |  |  |  |
| **ν_2_/ν_1_** | **3525** | **3478** | **3370** |

| **Sign multiplication (synchronous × asynchronous)** | | | |
| --- | --- | --- | --- |
| **3370** | **+** | **-** |  |
| **3478** | **-** |  |  |
| **3525** |  |  |  |
| **ν_2_/ν_1_** | **3525** | **3478** | **3370** |

**Table S3. Signs of the main cross-peaks in 2DCOS synchronous and asynchronous spectra from 1800-1520 cm^-1^.**

| **Cross-peak signs (left: synchronous; right: asynchronous)** | | | | |
| --- | --- | --- | --- | --- |
| **1570** | **-, -** | **-, -** | **+, -** |  |
| **1637** | **-, -** | **+, -** |  |  |
| **1726** | **-, -** |  |  |  |
| **1755** |  |  |  |  |
| **ν_2_/ν_1_** | **1755** | **1726** | **1637** | **1570** |

| **Sign multiplication (synchronous × asynchronous)** | | | | |
| --- | --- | --- | --- | --- |
| **1570** | **+** | **+** | **-** |  |
| **1637** | **+** | **-** |  |  |
| **1726** | **+** |  |  |  |
| **1755** |  |  |  |  |
| **ν_2_/ν_1_** | **1755** | **1726** | **1637** | **1570** |

The sequential spectral variations were determined according to Noda’s rule, a well-established criterion in two-dimensional correlation spectroscopy. When ν_1_ > ν_2_, if the corresponding synchronous and asynchronous cross-peaks show the same sign, the spectral feature at the higher wavenumber ν_1_ changes earlier. Conversely, if the signs are opposite, the spectral feature at the lower wavenumber ν_2_ changes earlier. In the notation “A → B”, A represents the component that responds earlier than B during the heating process.The complete cross-peak sign analysis and the derived response sequences for the O–H stretching region and the C=O/C=N region are summarized in Tables S2 and S3, respectively.

**Table S4. Adhesion strength compared with reported gel/ionogel** materials**.**

| **Material** | **Adhesion strength (kPa)** | | | | **Ref.** |
| --- | --- | --- | --- | --- | --- |
|  | Paper | Metal | Glass | Plastic |  |
| SBMA/MAA/SA hydrogels | - | 40.52 | 20.08 | - | [1] |
| BA/[EMIM][NTf_2_] ionogel | - | ≈35 | ≈35 | - | [2] |
| **LCPAD hydrogels** | 30.3 | 25.3 | 28.1 | 23.2 | [3] |
| **PN4 eutectogel** | 13.2 | 5.5 | 2.6 | 4.4 | [4] |
| **TPU nanonet/AA/**  **[BMIM]BF_4_**] ionogel | - | - | 23 | - | [5] |
| **MEA/[VBT][TFSI] ionogel** | - | 40.46 | 47.06 | - | [6] |
| **PAGDP-Ag hydrogel** | - | 6.2 | 9.8 | 8.2 | [7] |
| **Gly-lignin ionogel** | - | 21.8 | - | 23.5 | [8] |
| PCGK-CT hydrogel | - | 4.14 | - | - | [9] |
| **P(BZMA-co-HEA)/IL** | **57.40** | **53.64** | **42.41** | **39.98** | **This work** |

**Table S5. Compared with other reported flexible temperature sensors.**

| **Material** | **TCR(%/℃)** | **Temperature range (℃)** | **Ref.** |
| --- | --- | --- | --- |
| **MCS** | **-2.02** | **30-50** | [10] |
| **CPPFe@TA** | **-1.89** | **25-40** | [11] |
|  | **-0.44** | **40-70** |  |
| **APCG** | **-1.7** | **10-50** | [12] |
| **CLPRGG** | **-1.24** | **25-55** | [13] |
|  | **-0.61** | **55-80** |  |
| **PSMP/Gly** | **-1.184** | **20-80** | [14] |
| **PAA-SCMC** | **-0.88** | **20-60** | [15] |
| **PVA-nano silica-glycerol** | **-0.24** | **20-50** | [16] |
| **P(BZMA-co-HEA)/IL** | **-2.93** | **25-50** | **This work** |
|  | **-1.2** | **50-65** |  |

**Table S6. Comprehensive performance comparison of recently reported ionogels/hydrogels for wearable strain sensing applications.**

| **Material** | **Stretchability** | **Adhesion strength** | **Weight**  **retention** | **Swelling ratio** | **Sensing performance (range; GF)** | **Durability** | **Environmental adaptability** | **Ref.** |
| --- | --- | --- | --- | --- | --- | --- | --- | --- |
| PSA-DA/DES | 1600% | 199 kPa | ≈76%  (15 days) | 3% | 0-500%;  3.9 | 300 cycles at 100% | air/underwater | [17] |
| N_4_T_1_IS | ~450% | 24.80 kPa | 98.96 %  (>90 days) | ≈-40% | 0-400%;  4.5 | 2000 cycles at 50% | air/underwater | [18] |
| TPU -(HFBA-HEA)/  [EMIM][TFSI] | ~400% | 102.7 ± 2.8 μJ cm^-2^ | >80%  (>15 days) | ≈-20% | 0-400%;  3.1 | 3000 cycles at 50% | air | [19] |
| PCCE | ~250% | NR | > 85%  (>7 days) | <25% | 0-250%; 3.73 | 200 cycles at 50% | air/-20 ℃ | [20] |
| S-PM | 558% | NR | NR | 75% | 0-200%; 0.73 | 500 cycles | air | [21] |
| IL-PS | 635% | 73.51 kPa | NR | 3% | 0-533%; 1.54 | 200 cycles at 50% | air/underwater | [22] |
| MCS | 704% | 12.9 kPa | ≈100%  (15 days) | -10.2% | 0-680%; 1.134 | NR | air/50 ℃/  underwater | [23] |
| AL-CNF/PAA/  PVA-EG | >300% | 42 N/m | NR | 10% | 0-600%; 5.13 | 100 cycles at 50% | -25.88 °C/  underwater | [24] |
| P(AA-PEA_X_)-  Zr^4+^ | 364.31% | NR | NR | -2.49% | 0-200%; 2.42 | 1000 cycles at 10% | underwater | [25] |
| PTPAVI-Al^3+^ | 854% | 16.49 kPa | >70%  (15 days) | -1.2% | 0-800%; 2.01 | 1200 cycles at 30% | air/-32.34 ℃/  underwater | [26] |
| CMTT | 1240% | NR | NR | 44% | 0-200%; 17.4 | 1000 cycles at 50% | air/underwater | [27] |
| PSZC | 689% | NR | NR | 1.7% | 0-600%; 2.13 | 500 cycles at 100% | air/underwater | [28] |
| EHB | 798% | NR | NR | 1.7% | 0-700%; 1.501 | 3000 s at 100% | air/underwater | [29] |
| HSG | 1191% | 572.3 kPa | NR | 1.5% | 0-300%;  1.88 | 60 cycles at 50% | air/underwater | [30] |
| PAHCT | 798% | 59.1 kPa | NR | 5.5% | 0-500%;  1.13 | 800 cycles at 100% | air/underwater | [31] |
| **P(BZMA-co-**  **HEA)/IL** | 1100%, | 57.4 kPa | 98.86% at RT  (30 days)  95.25% at 65 ℃  (30 days) | 2.94% | 0-700%;  3.22 | 1000 cycles at 100% | air/65 ℃/  underwater | This work |

[1] F. Han, S. Chen, F. Wang, M. Liu, J. Li, H. Liu, Y. Yang, H. Zhang, D. Liu, R. He, W. Cao, X. Qin, F. Xu, *Adv. Sci.* **2025**, *12*, 2412726.

[2] S. Wang, S. Liu, J. Zhang, Z. Yu, Y. Shao, C. Zhao, J. Ma, X. Fu, M. Yang, L. Chen, M. Liu, L. Wen, *Adv. Sci.* **2025**, *12*, 2414761.

[3] Y. Mi, T. Wu, Y. Sun, Y. Wang, X. Cao, N. Wang, *Adv. Funct. Mater.* **2026**, *36*, e74956.

[4] C. Yang, B. Xu, Y. Tang, Z. Liu, H. Mi, F. Guo, X. Liao, J. Qiu, *Adv. Funct. Mater.* **2026**, *36*, e07051.

[5] Y. Xu, X. Jing, P. Feng, M. F. Antwi‐Afari, Y. Liu, F. Liu, S. Li, H. Mi, *Adv. Funct. Mater.* **2026**, *36*, e17882.

[6] X. Wang, K. Ma, C. Wang, H. Zheng, S. Yu, K. Chen, S. Qiao, *Adv. Funct. Mater.* **2026**, *36*, e22323.

[7] Z.-C. Xu, Y.-Q. Yang, X.-W. Pang, Y.-T. Xu, L.-X. Gong, L.-C. Tang, S.-N. Li, *J. Mater. Sci. Technol.* **2025**, *237*, 10.

[8] Z. Ye, H. Yu, H. Xie, W. Zhu, S. Shi, C. Liu, Y. Wang, J. Liao, Q. Sun, D. Zhao, X. Shen, *Adv. Sci.* **2025**, *12*, e06901.

[9]	Z. Yu, Y. Gu, Y. Ren, H. Ding, D. Wu, Z. Wu, J. Mou, *Microsyst. Nanoeng.* **2026**, *12*, 107.

[10] Y. Zhang, Y. Sun, J. Yang, R. Tian, J. Liu, X. Tang, J. Wang, Q. Li, *J. Mater. Chem. A* **2024**, *12*, 33734.

[11] X. Dang, Y. Fu, X. Wang, *Adv. Funct. Mater.* **2024**, *34*, 2405745.

[12] Y. Li, X. Yang, Y. Ding, H. Zhang, Y. Cheng, X. Li, J. Sun, Y. Liu, Y. Li, D. Fan, *Small* **2025**, *21*, 2411046.

[13] Y. Zou, Z. Liao, R. Zhang, S. Song, Y. Yang, D. Xie, X. Liu, L. Wei, Y. Liu, Y. Song, *Carbohydr. Polym.* **2025**, *348*, 122788.

[14] W.-Y. Guo, Y.-T. Jiang, B. Wang, M.-G. Ma, *J. Mater. Sci. Technol.* **2026**, *246*, 299.

[15] J. Wei, C. Liu, L. Shi, Y. Liu, H. Lu, *Carbohydr. Polym.* **2025**, *350*, 122943.

[16] Y. Liu, C. He, J. Fang, J. Zhou, K. Shang, X. Pu, L. Qian, T. Yang, *Mater. Today Phys.* **2024**, *41*, 101331.

[17] Z. Zhang, A. Yao, P. Raffa, *Adv. Funct. Mater.* **2024**, *34*, 2407529.

[18] X. Tang, J. Liu, R. Tian, Y. Zhang, X. Wang, L. Zhang, Z. Li, J. Yuan, Y. Jiang, Y. Sun, *Chem. Eng. J.* **2026**, *529*, 172746.

[19] J. Zhang, Z. Ma, M. Li, M. Lou, H. Wang, L. Jia, *Adv. Funct. Mater.* **2025**, *35*, 2415694.

[20] H. Pan, J. Huang, X. Huang, Y. Liang, N. Shen, L. Kong, L. Fan, R. Jin, *Chem. Eng. J.* **2025**, *520*, 166097.

[21] S. Zhang, F. Guo, X. Gao, M. Yang, X. Huang, D. Zhang, X. Li, Y. Zhang, Y. Shang, A. Cao, *Adv. Sci.* **2024**, *11*, 2405880.

[22] L. Han, C. Shan, M. Cui, R. Su, R. Huang, *Small* **2025**, *21*, e06902.

[23] Y. Zhang, Y. Sun, J. Yang, R. Tian, J. Liu, X. Tang, J. Wang, Q. Li, *J. Mater. Chem. A* **2024**, *12*, 33734.

[24] X. Gao, Y. Wang, J. Wu, Z. Xue, C. Yan, H. Zhang, Y. Wang, Y. Zhang, M. Jiang, Y. Zhao, *ACS Sustain. Chem. Eng.* **2024**, *12*, 15159.

[25] S. Pan, C. Chang, G. Wu, Z. He, C. Zhang, S. Xiong, G. Tong, X. Zhu, *ACS Appl. Mater. Interfaces* **2025**, *17*, 9760.

[26] T. Lei, X. Duan, H. Zhao, S. Ma, X. Ma, N. Wang, Q. Zhang, A. Wan, Z. Xia, W. Shou, J. Fan, *Chem. Eng. J.* **2025**, *504*, 158700.

[27] H. Wang, X. Lin, X. Li, D. Lv, J. Zhang, L. Wei, J. Tang, Y. Lin, X. Wu, X. Xu, *Small* **2025**, *21*, 2503067.

[28] Y. Zhang, X. Jing, J. Zou, P. Feng, G. Wang, J. Zeng, L. Lin, Y. Liu, H. Mi, S. Nie, *Adv. Funct. Mater.* **2024**, *34*, 2410698.

[29] R. He, X. Zheng, Y. Zhang, C. Yuan, Y. Xu, B. Zeng, L. Dai, *Matter* **2026**, *9*, 102707.

[30] R. Zhou, Y. Jin, W. Zeng, H. Jin, Y. Li, J. Mei, J. Liu, *Adv. Funct. Mater.* **2024**, *34*, 2316687.

[31] R. Wu, T. Zhu, Y. Ni, C. Wu, W. Wang, K. Zhao, J. Huang, Y. Lai, *Adv. Funct. Mater.* **2026**, *36*, e15120.
